# TRPM7 Deficiency Accelerates Vascular Senescence by Inhibiting H3K18 Lactylation

**DOI:** 10.1111/acel.70244

**Published:** 2025-10-02

**Authors:** Yue Wang, Jing Chen, Xuan Wang, Jin Li, Shujun Yang, Lingping Zhu, Zhenyu Li, Chuanchang Li, Wanzhou Wu, Yongping Bai

**Affiliations:** ^1^ Department of Geriatric Medicine Xiangya Hospital, Central South University Changsha People's Republic of China; ^2^ National Clinical Research Center for Geriatric Disorders Xiangya Hospital, Central South University Changsha People's Republic of China; ^3^ Department of Cardiology Xiangya Hospital, Central South University Changsha People's Republic of China

**Keywords:** H3K18, histone lactylation, TRPM7, vascular aging

## Abstract

Blood vessels exhibit a pronounced vulnerability to aging and are often at the forefront of systemic aging processes. Vascular endothelial cells, which line blood vessels and directly contact blood flow, are susceptible to damage and play a key role in vascular aging; however, the underlying mechanisms of their aging remain unclear. Here, we identify TRPM7 as a key molecule in vascular endothelial aging. Endothelial deletion of TRPM7 significantly accelerates premature vascular aging in mice. Mechanistically, TRPM7 deficiency reduces lactate production and inhibits the lactylation writer protein p300, leading to decreased histone H3K18 lactylation. This induces a gene expression profile reprogramming, increasing the expression of the senescence gene p21 and decreasing the expression of angiogenesis genes. Inhibiting p21 or supplementing lactate reversed premature vascular endothelial aging caused by TRPM7 deficiency. This study reveals the critical role of the TRPM7‐H3K18la axis in vascular aging, offering potential therapeutic targets for vascular anti‐aging interventions.

## Graphical abstract

TRPM7 deficiency reduces lactate production in endothelial cells and downregulates both p300 expression and nuclear translocation. These changes collectively lead to decreased H3K18 lactylation levels. Consequently, this downregulates genes vital for vascular homeostasis while upregulating genes associated with aging and vascular remodeling (including CDKN1A). These epigenetic alterations ultimately drive the aging process.

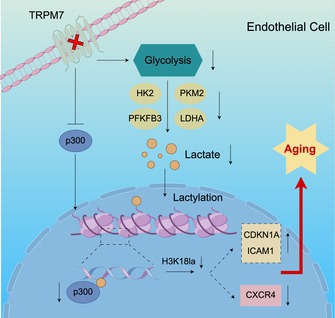

## 1 Introduction

Vascular aging represents a complex pathophysiological process characterized by progressive structural and functional deterioration of the arterial system (Cai et al. 2022; Hernandez‐Segura et al. 2018). This phenomenon encompasses endothelial dysfunction, arterial stiffening, reduced elasticity, and remodeling of the vascular extracellular matrix (ECM), driven by molecular and cellular alterations such as oxidative stress, chronic low‐grade inflammation, mitochondrial dysfunction, and cellular senescence (Ungvari et al. 2018). The vascular endothelium, a critical interface between blood and tissues, undergoes age‐related functional decline marked by impaired nitric oxide (NO) bioavailability, increased permeability, and pro‐inflammatory activation (Bloom et al. 2023). Concurrently, vascular smooth muscle cells (VSMCs) exhibit phenotypic switching, calcification, and reduced contractility, contributing to arterial rigidity (Bloom et al. 2023; Lacolley et al. 2017). The latest Human Protein Atlas indicates that vascular tissue is among the first organs to exhibit age‐related changes, beginning around age 50 and subsequently accelerating systemic aging (Y. Ding et al. 2025). These age‐associated changes collectively diminish vascular compliance and hemodynamic homeostasis, predisposing individuals to hypertension, atherosclerosis, stroke, and other cardiovascular diseases (CVDs) (Bloom et al. 2023). Alarmingly, epidemiological data reveal that over 60% of individuals aged 65+ exhibit subclinical vascular aging markers, with CVDs remaining the leading cause of mortality in aging populations globally (M. Jiang et al. 2024).

The clinical urgency to decipher vascular aging mechanisms stems from its dual role as both a biomarker and a driver of systemic aging. Unlike chronological aging, vascular aging is modifiable, offering therapeutic windows to delay age‐related morbidity (Grunewald et al. 2021; Mojiri et al. 2021). Current interventions—such as lifestyle modifications, antihypertensives, and statins—address downstream consequences but fail to target root mechanisms. Emerging evidence implicates dysregulated ion homeostasis, epigenetic modifications, and mechanotransduction pathways in vascular senescence, yet a unified mechanistic framework remains elusive (Y.‐N. Ding et al. 2018; Harraz and Jensen 2020; Maus et al. 2023; Venkatachalam 2022; J. Zhang et al. 2024). Elucidating these pathways is critical for developing precision therapies to preserve vascular resilience. For instance, targeting senescence‐associated secretory phenotype (SASP) components or enhancing DNA repair mechanisms has shown promise in preclinical models (Huang et al. 2023; Mehdizadeh et al. 2022; Uryga et al. 2016). However, the role of ion channels, particularly those integrating biochemical and mechanical signals, remains underexplored in vascular aging contexts. This knowledge gap underscores the need to investigate molecular hubs that orchestrate vascular homeostasis and their dysregulation during aging.

Transient receptor potential melastatin 7 (TRPM7), a unique member of the TRP channel superfamily, is a dual‐function protein possessing intrinsic ion channel and serine/threonine kinase activities (Nadler et al. 2001; Runnels et al. 2001). Structurally, it comprises a tetrameric cation‐permeable pore domain fused to a C‐terminal α‐kinase domain, enabling it to regulate cellular magnesium (Mg^2+^) and calcium (Ca^2+^) homeostasis while phosphorylating downstream targets (Duan et al. 2018). In vascular biology, TRPM7 is ubiquitously expressed in endothelial cells (ECs), VSMCs, and pericytes, where it modulates critical processes including cell proliferation, migration, apoptosis, and ECM remodeling (Clark et al. 2006; He et al. 2005; J. Jiang et al. 2007). Mechanistically, TRPM7 integrates mechanical forces (e.g., shear stress) and biochemical cues (e.g., oxidative stress and metabolism) to regulate vascular tone and barrier integrity (He et al. 2005; Schmitz et al. 2003; Yankaskas et al. 2021). Our and our partner's previous research demonstrated that TRPM7 promotes CRTC2 nuclear translocation and subsequent CREB1 phosphorylation via Ca^2+^/Calcineurin signaling (Wang et al. 2025; Wu et al. 2023). This, in turn, upregulates the transcription of glycolytic molecules such as HK2 and GLUT3, accelerating lactate production and proliferation in vascular endothelial cells (Wu et al. 2023). Importantly, the kinase domain of TRPM7 appears to have minimal impact on this process, highlighting the TRPM7 channel, rather than its kinase activity, as the primary structural component controlling endothelial cell glycolysis and lactate production (Wu et al. 2023). In tumor cells, silencing TRPM7 promotes HIF1α degradation and inhibits glycolysis by activating AMPK (Y. Chen et al. 2022). Despite these established roles, TRPM7's involvement in vascular aging remains enigmatic.

In this study, we found that TRPM7 regulates vascular aging. TRPM7 knockout reduces lactate levels and the lactylation writer p300 to decrease histone H3K18 lactylation, leading to increased expression of the senescence gene p21 and decreased expression of angiogenesis‐related genes. Lactate supplementation or p21 inhibition reversed premature vascular endothelial cell senescence caused by TRPM7 deficiency. This research enhances our understanding of TRPM7's role in vascular biology.

## 2 Results

### 2.1 The Lactylation of H3K18 Decreases in Aging Endothelial Cells

We established a replicative aging model by continuously passaging primary human umbilical vein endothelial cells (HUVECs). Cells isolated from the umbilical vein were designated as p0, and those passaged up to five times were considered young endothelial cells due to their consistent doubling time. After six passages (p6), the proliferation rate began to slow (Figure S1a). Therefore, cells passaged ten times (p10) were regarded as aged, showing significant changes in aging marker genes compared to young endothelial cells, including increased levels of p16INK4a and p21CIP1 and decreased levels of LaminB1 (Figure S1b,c).

**FIGURE 1:**
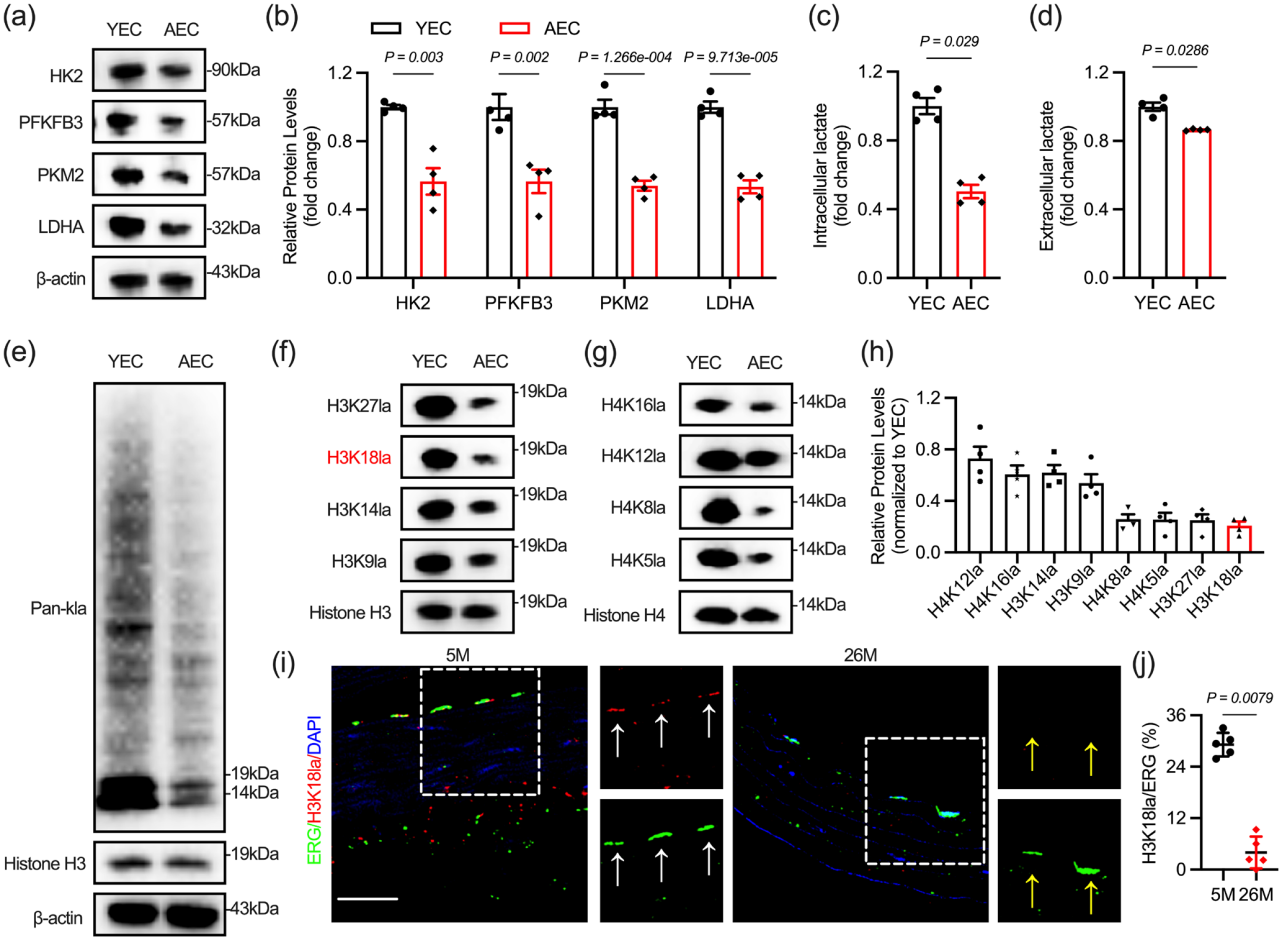
Decreased endothelial histone H3K18 lactylation in aging vessels. (a, b) The effect of aging on glycolytic enzyme protein levels in endothelial cells. (c, d) Lower Intracellular and extracellular lactate levels in AEC. (e) Less pan‐lactylation in AEC. (f–h) H3K18la exhibits the greatest decrease in AECs. (i, j) Decreased H3K18la in aortic endothelium of aged mice (26‐month‐old). Scale bar: 20 μm in (i). All differences in this figure are tested using Mann–Whitney tests.

Previous studies indicate that glycolysis in aging vascular endothelial cells is weakened (Hu et al. 2023). Thus, we examined several metabolic enzymes related to endothelial glycolysis and found that HK2, PFKFB3, and LDHA levels decreased in aging endothelial cells, accompanied by reduced intracellular lactate production and lower extracellular lactate levels (Figure 1a–d). Changes in intracellular lactate levels can lead to alterations in protein lactylation, significantly impacting cellular function (Xiaolu Li et al. 2022). Immunoblotting shows a reduced overall level of protein lactylation in aged endothelial cells, with the most distinct bands appearing around 15 and 10 kDa, typical positions for histones H3 and H4 (Figure 1e). In contrast, changes in protein acetylation are not significant (Figure S2). Histone lactylation, the earliest identified form of protein lactylation, regulates gene transcription by modulating chromatin accessibility (D. Zhang et al. 2019). Some studies have explored the relationship between histone lactylation and aging (J. Chen et al. 2025; Xiang Li et al. 2025; Liu et al. 2024). Recently, a study found that H4K12 lactylation in vascular smooth muscle is associated with vascular aging, but this has not yet been investigated in endothelial cells (Xuesong Li et al. 2024). We examined the known lactylation of histones H3 and H4 and found a significant decrease in H3K18 lactylation in aging endothelial cells (Figure 1f–h). We examined changes in H3K18 lactylation in the aortas of mice aged 5 and 26 months, finding a reduction in H3K18 lactylation in the nuclei of aortic endothelial cells in older mice (Figure 1i,j).

### 2.2 TRPM7 Is Reduced in Aging Endothelium

**FIGURE 2:**
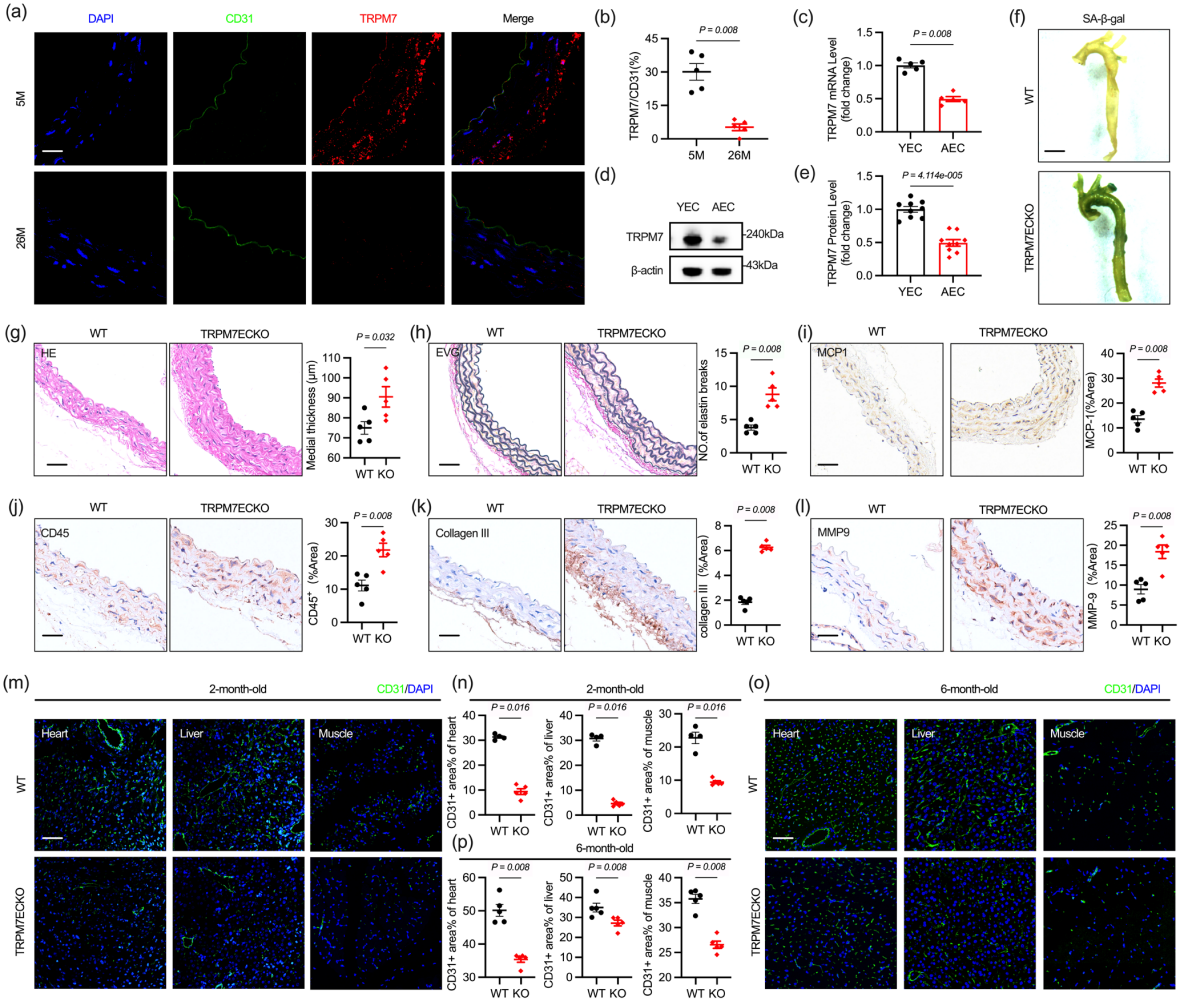
Endothelial TRPM7 deficiency accelerates vascular aging in young male mice. (a, b) TRPM7 expression is reduced in the aortic endothelium of aged mice. (c–e) TRPM7 transcription and protein levels are reduced in AECs in vitro. (f) Aortas from TRPM7 endothelial knockout male mice (TRPM7ECKO) were stained with SA‐β‐gal staining. (g) Aortic thickness was assessed in male mice using HE staining of the aorta. (h) EVG staining was used to evaluate aortic elastic fibers. (i, j) Higher MCP1 and CD45 staining indicates increased vascular inflammation in TRPM7ECKO male mice. (k, l) Increased Collagen III and MMP9 suggest vascular remodeling in aortas of TRPM7ECKO male mice. (m–p) TRPM7ECKO male mice exhibit reduced vascular endothelium in the heart, liver, and muslce at both 2 and 6 months of age. Scale bar: 20 μm in (a), 2 mm in (f), 40 μm in (g–l), 200 μm in (m and o). All differences in this figure are tested using Mann–Whitney tests.

Our previous research identified TRPM7 as a key regulator of glycolytic metabolism in endothelial cells. It induces Ca^2+^ influx, activating calcineurin, which subsequently leads to the dephosphorylation and nuclear localization of CRTC2. This process results in the phosphorylation of CREB1, promoting the transcriptional regulation of GLUT3 and facilitating glucose uptake and metabolism in endothelial cells, thereby playing a role in angiogenesis regulation (Wu et al. 2023). However, it remains unclear whether TRPM7 is involved in endothelial aging. Immunofluorescence revealed a decrease in TRPM7 expression in the aortic endothelium of aged mice (Figure 2a,b). An in vitro replicative aging model also confirmed the downregulation of TRPM7 protein and transcription levels during aging (Figure 2c–e).

### 2.3 TRPM7 Deficiency Accelerates Vascular Senescence in Mice

We deleted Trpm7 in mouse endothelium (TRPM7ECKO) to examine its impact on aging. Aortic SA‐β‐gal staining was intensified in 6‐month‐old Trpm7 endothelial knockout male mice (Figure 2f). Pathological analysis revealed thicker aortic walls and increased elastic fiber fragmentation in TRPM7ECKO male mice (Figure 2g,h). Elevated levels of MCP1 and the inflammatory cell marker CD45 are also present in the aorta of TRPM7ECKO mice (Figure 2i,j). Increased levels of type III collagen and matrix metalloproteinase 9 (MMP9) were observed in the aorta of TRPM7ECKO mice (Figure 2k,l). Furthermore, our previous research indicates vascular developmental abnormalities in the retinas of neonatal TRPM7ECKO mice (Wu et al. 2023). Surprisingly, we observed a reduced number of endothelial cells in the hearts, livers, and muscles of 2‐month‐old and 6‐month‐old TRPM7ECKO mice (Figures 2m–p, S3a–g), suggesting a sparse vascular network. Deletion of TRPM7 in endothelial cells has no effect on blood pressure in 6‐month‐old mice (Figure S3i). Notably, similar phenotypes were observed in 6‐month‐old female TRPM7 endothelial knockout mice (Figure S4a–h). In summary, these results indicate that endothelial defects in Trpm7 result in fewer organ microvessels, increased vascular inflammation, and vascular remodeling, all of which are characteristic phenotypes of vascular senescence.

### 2.4 TRPM7ECKO Mice Showed Premature Aging in Multiple Organs

Previous studies have reported that vascular senescence can trigger organ senescence (Grunewald et al. 2021; Mojiri et al. 2021). We investigated whether endothelial Trpm7 deficiency leads to senescence in other organs by isolating various organs from mice and performing SA‐β‐gal staining. The results showed that the liver, kidneys, muscles, and brain of TRPM7ECKO mice exhibited more intense staining compared to controls (Figures S5a–c, S6a–c). However, there was little visible difference in the adipose and heart tissues (Figures S5b, S6b). These findings suggest that endothelial Trpm7 deficiency can affect the senescence of certain organs in mice.

### 2.5 TRPM7 Knockout Inhibits Angiogenesis Following Hindlimb Ischemia

**FIGURE 3:**
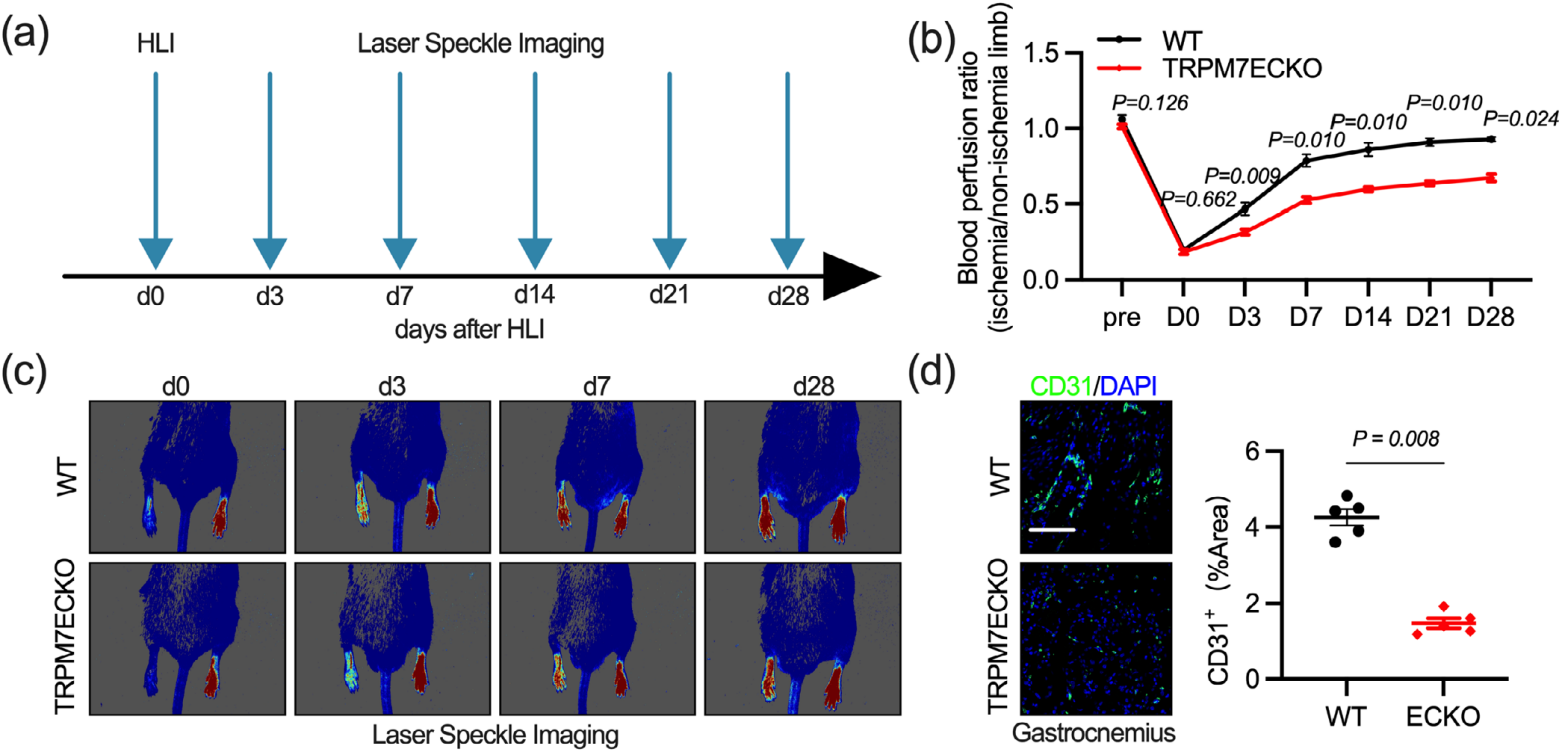
Endothelial TRPM7 deficiency impairs hindlimb muscle angiogenesis following ischemia. (a–c) Laser Doppler was used to assess the temporal recovery of foot perfusion in 2‐month‐old mice following femoral artery ligation. (d) Immunofluorescence was used to assess gastrocnemius muscle vascular endothelial coverage on day 7 post‐femoral artery ligation. Scale bar: 100 μm in (d). All differences in this figure are tested using Mann–Whitney tests.

Vascular senescence often results in a reduced response to stress in blood vessels. We developed a hindlimb femoral artery ligation model to investigate whether endothelial‐specific Trpm7 knockout‐mediated vascular senescence affects hypoxia‐induced angiogenesis (Figure 3a). Laser Doppler analysis indicates that Trpm7ECKO mice exhibit delayed blood flow recovery (Figure 3b,c). This may be due to the lower density of microvasculature in the muscles (Figure 3d). These results demonstrate that Trpm7 knockout impairs the vascular bed's ability to respond to hypoxia.

### 2.6 Inhibition of TPRM7 Accelerates Endothelial Cell Senescence In Vitro

**FIGURE 4:**
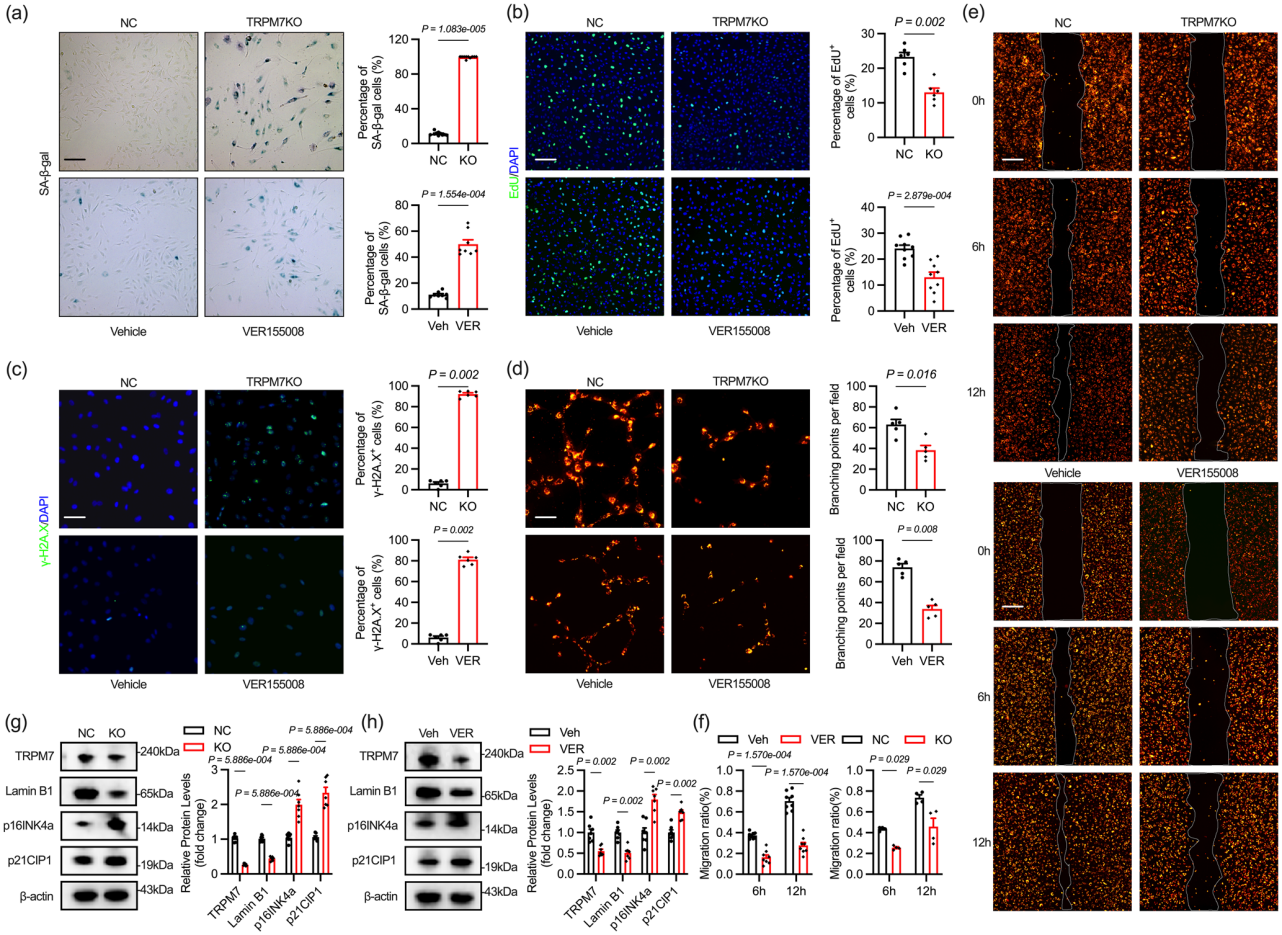
TRPM7 inhibition impairs endothelial function and accelerates aging in vitro. (a) The effect of TRPM7 knockout or pharmacological inhibition on SA‐β‐gal staining in YEC. (b) TRPM7 deficiency inhibits YEC proliferation. (c) TRPM7‐deficient YECs exhibit an elevated rate of γ‐H2A.X positivity. (d) TRPM7 inhibition reduces YEC tube formation. (e, f) Impaired TRPM7 reduces cell migration. (g, h) The impact of impaired TRPM7 on genes associated with cellular senescence. Scale bar: 300 μm in (a, b, e), 200 μm in (d), 120 μm in (c). All differences in this figure are tested using Mann–Whitney tests.

We subsequently clarified the impact of TRPM7 deficiency on endothelial cell senescence. We used CRISPR/Cas9 to knock out TRPM7 or pharmacological inhibition of TRPM7 by VER155008 in young HUVECs, resulting in increased SA‐β‐gal staining (Figures 4a, S7). Reduced EdU and increased γH2AX levels were observed, indicating that TRPM7 inhibition results in decreased cell proliferation and increased DNA damage (Figure 4b,c). TRPM7 inhibition also impairs cell migration and tube formation abilities (Figure 4d–f). The expression of senescence marker genes p16INK4a and p21CIP1 is elevated in TRPM7 inhibited cells, while Lamin B1 is reduced, consistent with the changes observed in senescent endothelial cells (Figure 4g,h).

### 2.7 TRPM7 Deficiency Suppresses Endothelial Cell Glycolysis and H3K18 Lactylation

**FIGURE 5:**
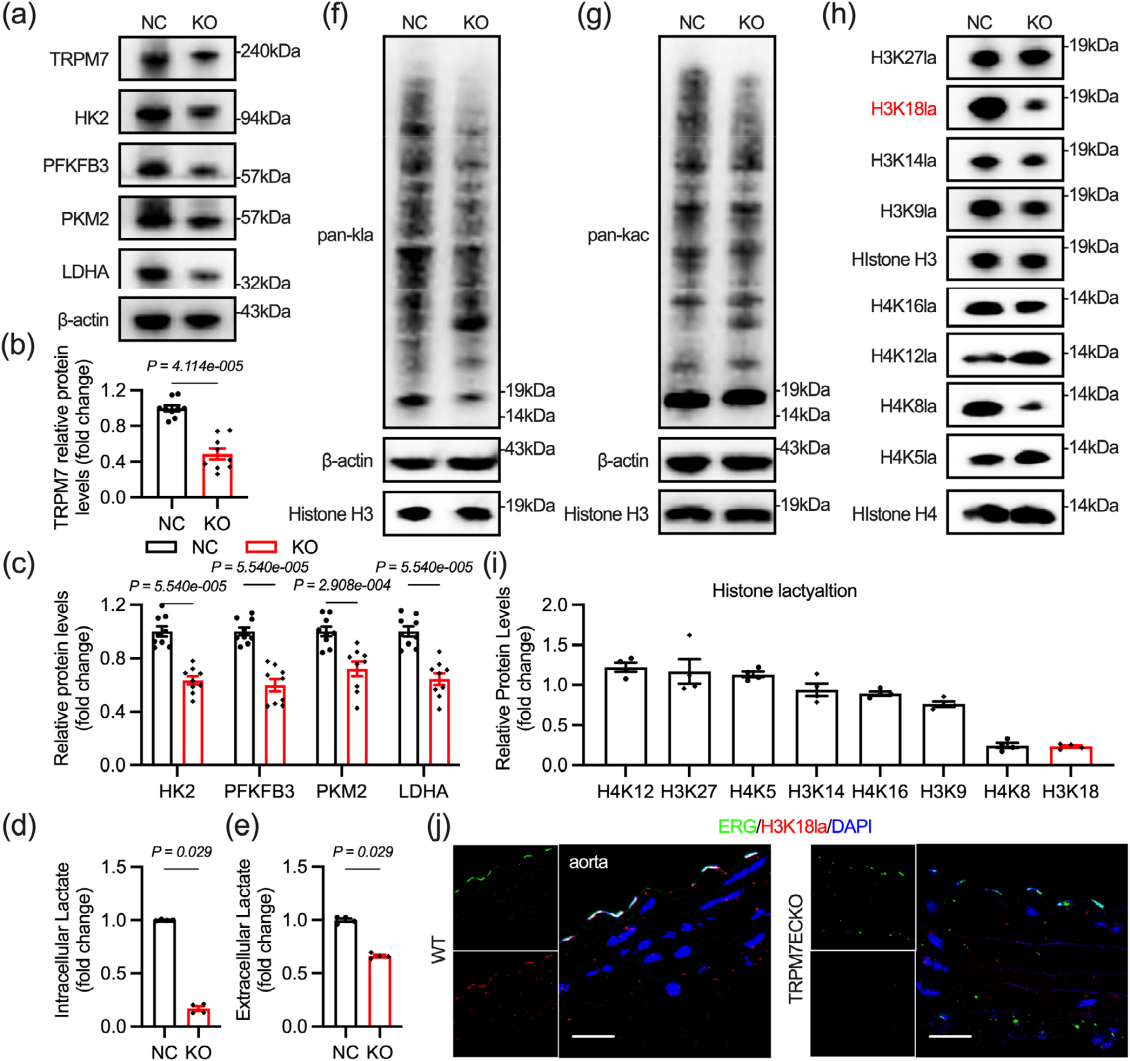
TRPM7 deficiency inhibits histone H3K18 lactylation in vitro and vivo. (a–c) TRPM7 knockout reduces the protein levels of glycolytic enzymes. (d, e) TRPM7 knockout inhibits lactate production. (f, g) Effects of TRPM7 knockout on global protein lactylation and acetylation. (h, i) TRPM7 knockout results in the strongest suppression of histone H3K18 in vitro. (j) Reduced H3K18 lactylation in the aortic endothelium of 2‐month‐old TRPM7 endothelial knockout mice. Scale bar: 20 μm in (j). All differences in this figure are tested using Mann–Whitney tests.

In previous research, we used shRNA to knock down TRPM7 and confirmed that this led to a decrease in glycolysis (Wu et al. 2023). Here, we employed CRISPR/Cas9 and VER to knockout or inhibit TRPM7 and observed the effects of different blocking techniques on endothelial glycolysis. TRPM7 knockout results in the downregulation of HK2, PFKFB3, PKM2, and LDHA, leading to reduced intracellular and extracellular lactate (Figure 5a–e). Surprisingly, unlike the general downregulation of histone H3 and H4 lactylation in aging endothelial cells, TRPM7 knockout primarily affects the lactylation of histone H3 (15 kDa) with limited impact on H4 lactylation (10 kDa) and pan‐kac (Figure 5f,g). Subsequently, we found that TRPM7 significantly inhibited H3K18 lactylation, consistent with aging endothelium (Figure 5h,i). The H3K18 lactylation level in aortic endothelial cell nuclei of TRPM7ECKO mice was significantly reduced (Figure 5j). Pharmacological inhibition of TRPM7 in vitro fully replicates the significant suppression of H3K18 observed with TRPM7 knockout (Figure S8a–i).

Furthermore, lactate addition reversed the impaired H3K18 lactylation and endothelial cell function caused by TRPM7 inhibition, suggesting that TRPM7 may regulate H3K18 lactylation by reducing intracellular lactate levels (Figures S9a–f, S10a–f).

### 2.8 TRPM7 Deficiency Reduces the Expression and Nuclear Localization of the Lactate Modification Writer p300

**FIGURE 6:**
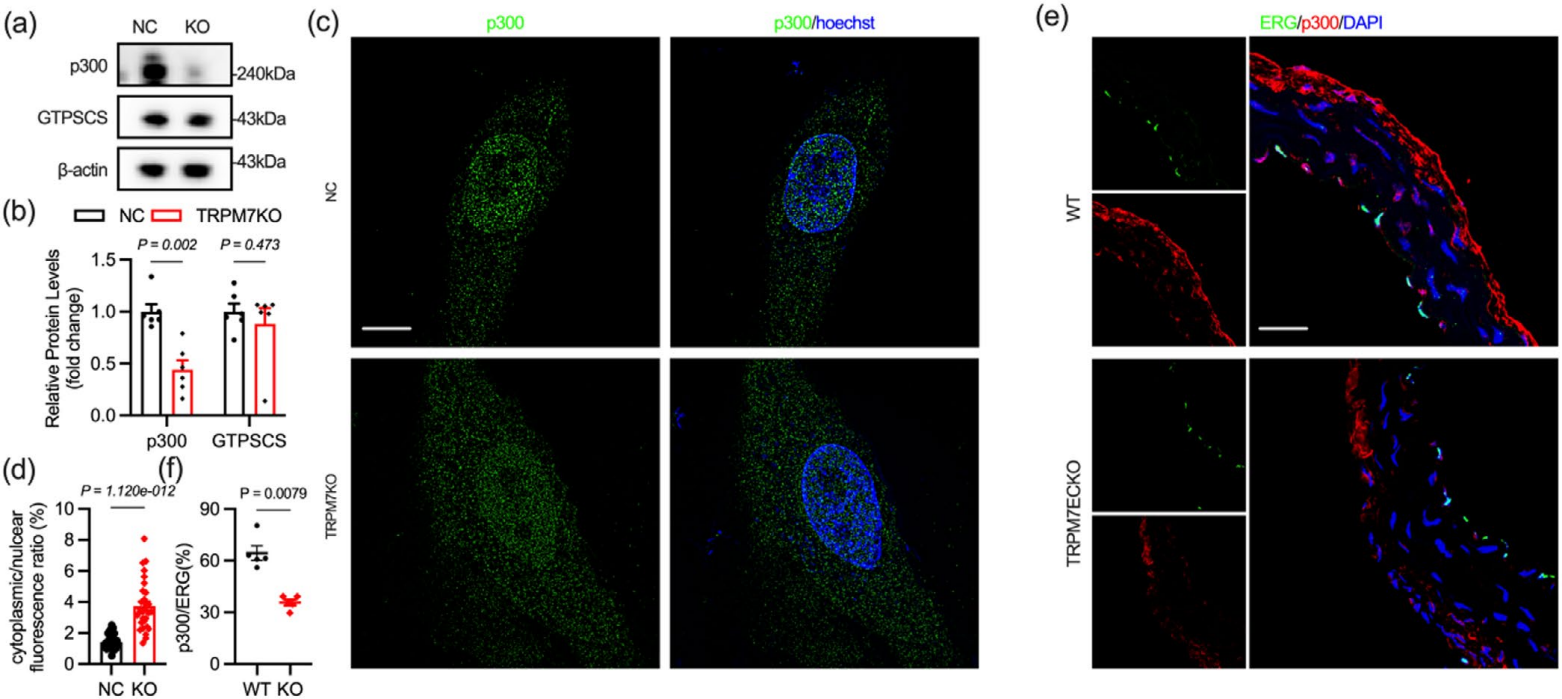
TRPM7 knockout reduces p300 expression and nuclear translocation. (a, b) Effects of TRPM7 knockout on endothelial cell p300 and GTPSCS protein expression. (c, d) Effect of TRPM7 knockout on the ratio of nuclear p300 localization in endothelial cells. (e, f) p300 expression levels in the nuclei of vascular endothelial cells (ERG+) from TRPM7ECKO and wild‐type mice. Scale bar: 10 μm in (c), 150 μm in (e). All differences in this figure are tested using Mann–Whitney tests.

Protein lactylation is regulated by diverse writers and erasers. p300 and GTPSCS are recently identified writers with a high preference for H3K18 lactylation (Dong et al. 2024; Ji et al. 2025; Liu et al. 2025). TRPM7 knockout inhibits p300 expression and nuclear localization in endothelial cells in vitro, processes essential for p300‐mediated histone H3K18 lactylation (Figure 6a–d). TRPM7, however, remains unaffected by GTPSCS (Figure 6a). In vivo, p300 was significantly reduced in the endothelium of TRPM7ECKO mice, with decreased signal overlap in endothelial cell nuclei (Figure 6e,f). Pharmacological inhibition of TRPM7 also reduces p300 protein levels and nuclear localization, suggesting that endothelial TRPM7 deficiency suppresses p300 expression and function (Figure S11a–d).

### 2.9 TRPM7 Channels, Not Its Kinase Activity, Are Required for Regulating H3K18 Lactylation

VER155008, a selective TRPM7 channel inhibitor, significantly reduces TRPM7‐mediated changes in membrane potential and strongly inhibits H3K18 histone lactylation (Rössig et al. 2022). Therefore, we hypothesize that TRPM7's channel activity is crucial for its regulation of H3K18 lactylation. Subsequently, we rescued TRPM7 channel pore mutants (TRPM7PM) in TRPM7‐knockout endothelial cells, where these mutants reportedly lack channel activity but retain kinase structure (Krapivinsky et al. 2006). The results showed that TRPM7PM failed to rescue the functional defects and impaired H3K18 lactylation in TRPM7KO endothelial cells, suggesting that the channel activity of TRPM7 is necessary for H3K18 lactylation (Figure S12a–f).

### 2.10 TRPM7 Knockout Promotes Endothelial Aging Gene Expression by Suppressing H3K18 Lactylation

To identify the biological processes influenced by TRPM7 through H3K18 lactylation, we conducted CUT‐tag and RNA‐seq analyses on TRPM7 knockout endothelial cells and control groups, followed by a combined analysis. Transcriptome Gene Set Enrichment Analysis (GSEA) revealed that TRPM7 knockout resulted in the activation of p53, NF‐κB, and TNF‐α signaling pathways (Figure S13a–g). GO and KEGG analyses suggest that TRPM7‐induced downregulated genes are associated with “blood vessel development”, “blood vessel morphogenesis”, and “cardiovascular development”, while upregulated genes are associated with “stress response”, “respond to abiotic stimulus” (Figure S13c–g). Subsequently, we examined TNF‐α and NF‐κB activity, finding increased TNF‐α and NF‐κB expression in the aortas of TRPM7ECKO mice, which supports the inference of their activation (Figure S13h).

**FIGURE 7:**
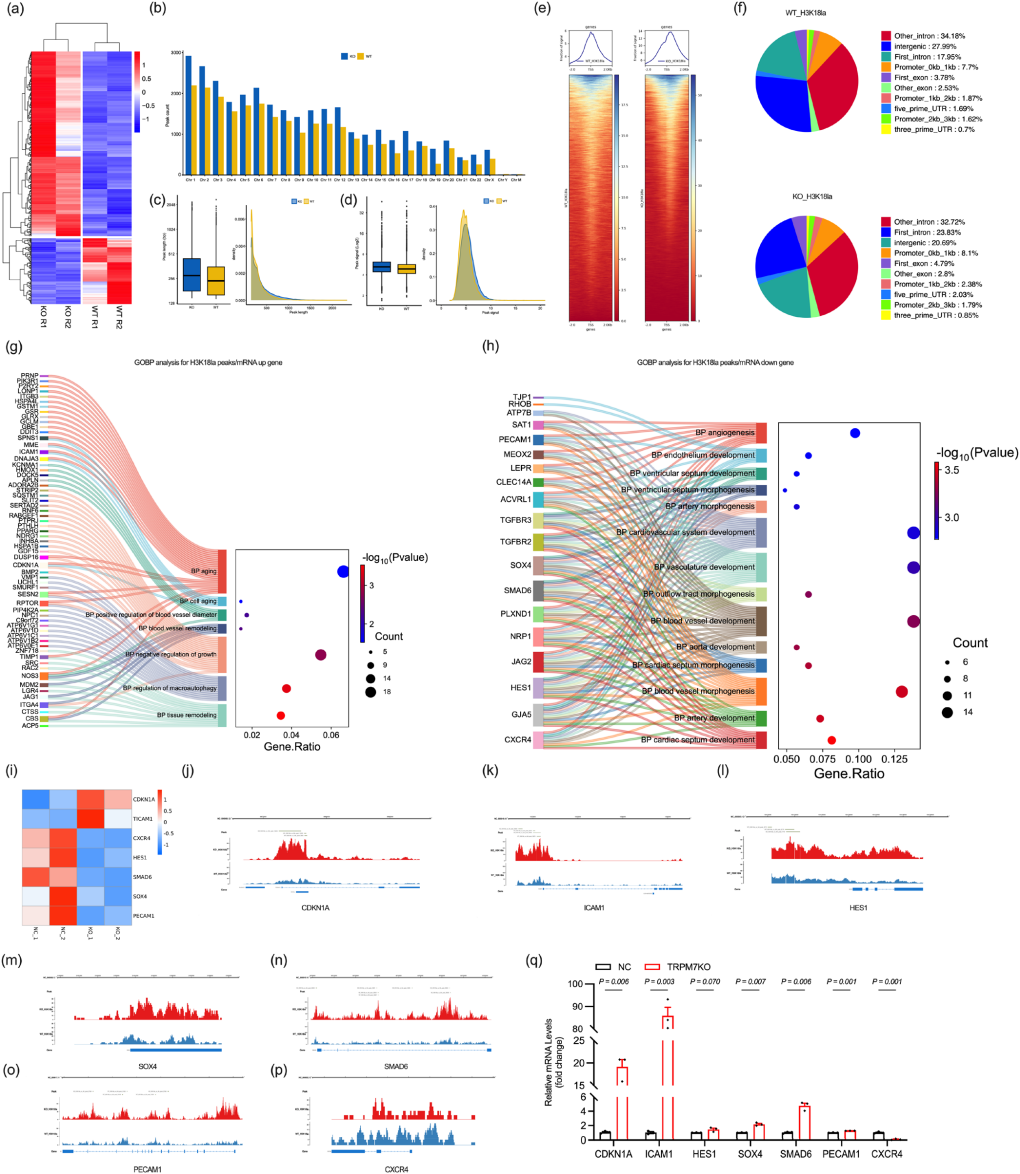
CUT&Tag and RNA‐seq analysis reveals TRPM7 knockout‐induced disruption of gene expression in endothelial cells. (a) Heatmap of altered H3K18la peaks associated with TRPM7KO. (b) Distribution of the H3K18la peaks across chromosomes. (c, d) The length and signals of H3K18la peaks. (e) Heatmaps and intensity profiles of H3K18la around ±2 kb of TSS throughout the genome. (f) Distribution of the altered H3K18la peaks in the genomic elements. (g, h) GO enrichment analysis was performed on differentially expressed genes that showed consistent trends in both mRNA‐seq and CUT&Tag data. (i–q) Validation of gene expression profiles related to differential aging and vascular homeostasis. All differences in this figure are tested using multiple unpaired Welch *t*‐test.

Combined analysis revealed that TRPM7 knockout cells exhibited increased chromatin accessibility and transcription in 368 genes, while both decreased in 132 genes (Figure 7a–f). GO analysis indicates that the upregulation of genes primarily pertains to “aging”, “cell aging”, “blood vessel remodeling”, “negative regulation of growth” (Figure 7g). The downregulated genes are primarily associated with “angiogenesis”, “endothelium development”, “artery morphogenesis” (Figure 7h). p21CIP1, encoded by the CDKN1A gene, is a key regulator of cellular senescence and is associated with the p53 signaling pathway (Pinto et al. 2017). Its upregulation promotes senescence. TRPM7 knockout significantly enhances CDKN1A expression levels via H3K18la downregulation, as well as ICAM1, PECAM1, and reduces arteriogenesis key gene CXCR4 (Figure 7i–q). These findings suggest that TRPM7 knockout may accelerate endothelial senescence by activating pathways such as p53/p21 and TNFα, potentially through the suppression of histone H3K18 lactylation.

### 2.11 Eliminating p21CIP1 Reverses TRPM7KO‐Dependent Endothelial Senescence

**FIGURE 8:**
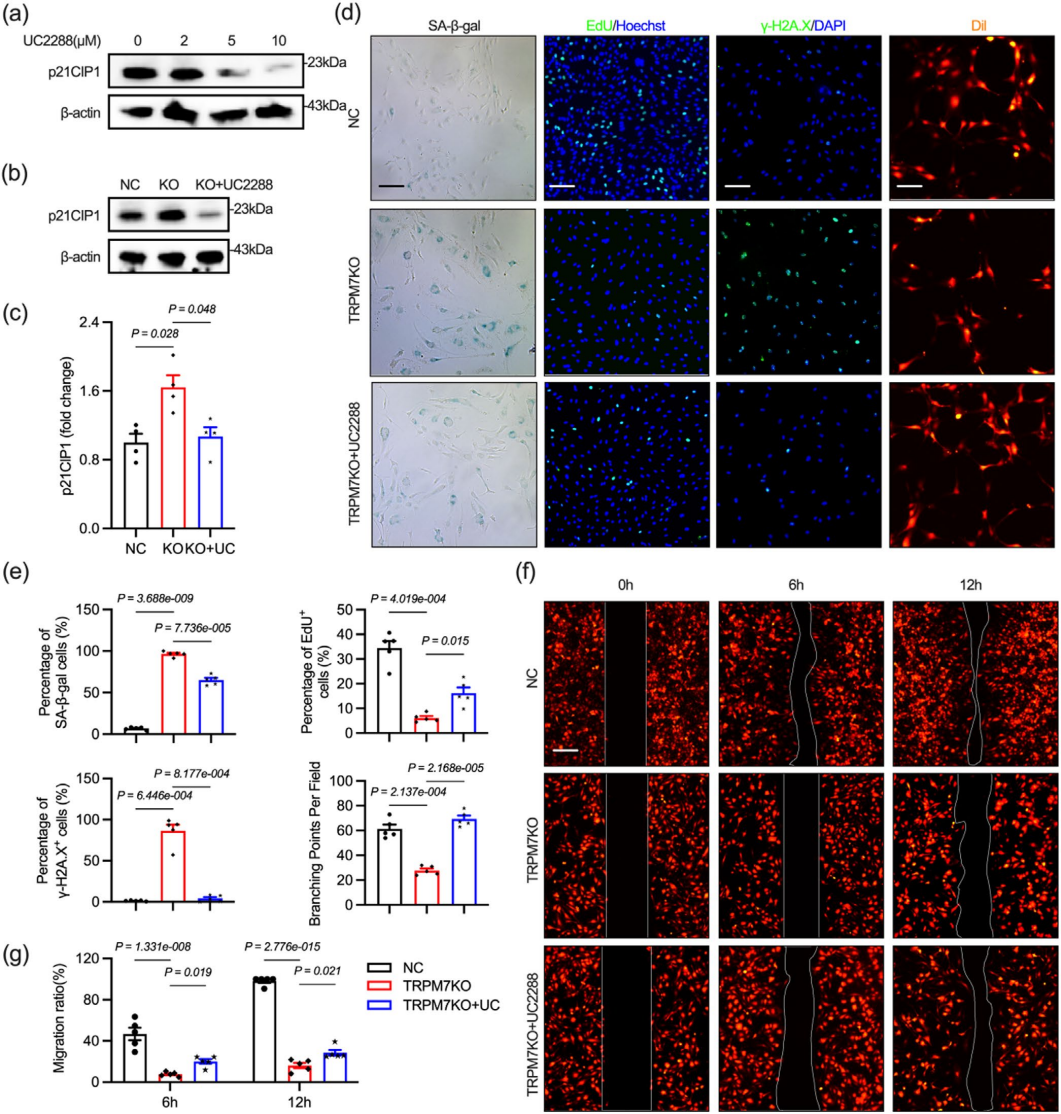
Eliminating p21CIP1 rescues premature vascular endothelial senescence caused by TRPM7 knockout in vitro. (a) UC2288 at varying concentrations affects p21CIP1 expression (24 h). (b, c) UC2288 (10 μM, 24 h) inhibits the increase in p21 levels caused by TRPM7 knockout. (d, e) UC2288 rescued TRPM7KO endothelial cells from premature senescence, proliferation defects, impaired tube formation, and DNA damage. (f, g) UC2288 rescued the migration of TRPM7KO endothelial cells. Scale bar: 300 μm in (f), 300 μm in SA‐β‐gal stanning and 200 μm in EdU and γ‐H2A.X and 120 μm in tubeformation. Differences in (c) are tested using Kruskal–Wallis test with Dunn's multiple correction and (e) are using Brown‐Forsythe and Welch ANOVA test with Dunnett's T3 multiple correction and (g) are using Two‐way ANOVA with Dunnett's multiple correction.

To determine if p21CIP1 is a critical downstream effector by which TRPM7 deficiency inhibits H3K18 lactylation‐mediated endothelial senescence, we used the p21CIP1 inhibitor UC2288 to deplete p21CIP1 in TRPM7KO cells (Figure 8a–c). The p21CIP1 inhibitors rescued premature endothelial cell senescence caused by TRPM7 deficiency, a finding replicated using a pharmacological TRPM7 inhibition model (Figures 8d–g, S14a–f). This suggests that the reduction of H3K18 lactylation‐dependent increase in p21 plays a crucial role in TRPM7 deficiency‐induced premature endothelial senescence.

## 3 Discussion

Reduced endothelial cell proliferation is a hallmark of vascular aging, leading to impaired angiogenesis and decreased organ perfusion. Enhancing endothelial proliferation and angiogenesis with VEGF can help delay aging and extend lifespan (Grunewald et al. 2021). Although TRPM7 was discovered over two decades ago, its role in endothelial cell proliferation remains contentious (Abidin et al. 2025). Some in vitro studies have reported that TRPM7 knockdown promotes endothelial cell proliferation (Baldoli et al. 2013; Fan et al. 2023; Inoue and Xiong 2009; Zeng et al. 2015; Zhou et al. 2020), while others have found the opposite effect (Baldoli and Maier 2012; Zhu et al. 2019). Unlike the contradictory phenomena observed in in vitro studies, TRPM7 knockdown in animals consistently results in impaired endothelial proliferation. In our previous research, we demonstrated that siRNA‐mediated knockdown of TRPM7 in endothelial cells impairs both their proliferation and migration (Wu et al. 2023). Additionally, neonatal mice with endothelial‐specific TRPM7 deficiency exhibit impaired retinal angiogenesis, providing evidence for TRPM7's role in endothelial cell proliferation (Wu et al. 2023). TRPM7 knockdown in zebrafish reduces vascular endothelial cell proliferation and impairs vascular repair after injury (Bowley et al. 2024). These transgenic animal studies highlight the beneficial role of TRPM7 in promoting endothelial cell proliferation. In this study, we employed CRISPR/Cas9 technology to knockout TRPM7 in primary vascular endothelial cells, revealing that this intervention inhibits cell proliferation and induces DNA damage. Similar effects were observed with the use of TRPM7‐specific inhibitors. In vivo, two‐month‐old TRPM7‐deficient mice showed impaired revascularization potential in response to ischemic stimuli, while at six months, they exhibited an accelerated multi‐organ aging phenotype. Collectively, these findings underscore the critical importance of TRPM7 in endothelial cell proliferation and support its role as a key determinant in vascular aging and regeneration.

TRPM7 is a unique channel‐enzyme that regulates downstream signaling pathways by modulating Ca^2+^ and Mg^2+^ homeostasis, while its kinase domain directly phosphorylates substrates to influence cellular functions (Desai et al. 2012; Dorovkov and Ryazanov 2004; Krapivinsky et al. 2014; Nadler et al. 2001; Runnels et al. 2001). In this study, we observed that TRPM7 deficiency led to a global reduction in histone lactylation, with the most pronounced decrease observed in H3K18 lactylation. TRPM7 knockout reshaped suppresses H3K18 lactylation, downregulating angiogenesis‐related genes such as CXCR4, HES1, SMAD6, SOX4, and PECAM1, while upregulating senescence‐associated genes like CDKN1A and ICAM1. This disrupted vascular homeostasis, promoting vascular and other organs senescence. Histone lactylation is primarily influenced by intracellular lactate levels, with additional regulatory factors including lactylation writer and eraser proteins. Our previous research demonstrated that TRPM7 deficiency suppresses GLUT3 transcription by inhibiting Ca^2+^ influx, thereby reducing glucose uptake and intracellular lactate levels in endothelial cells (Wu et al. 2023). Notably, neither Mg^2+^ nor the kinase domain could rescue the GLUT3 transcriptional impairment caused by TRPM7 deficiency (Wu et al. 2023). Rescuing TRPM7 mutants with an intact kinase structure but impaired channel function failed to reverse endothelial senescence, suggesting a possible redundant role for its kinase activity. This suggests that Ca^2+^ signaling‐mediated regulation of GLUT3 transcription may represent a potential mechanism linking TRPM7 to H3K18 lactylation, highlighting the critical role of the TRPM7‐Ca^2+^ axis in endothelial senescence. Furthermore, TRPM7‐deficient endothelial cells exhibit decreased expression and nuclear localization of p300, the highly selective H3K18 lactylation writer (R. Liu et al. 2025). This suggests that TRPM7 may mediate H3K18 lactylation via a “double‐hit” mechanism: by both reducing lactate production and impairing p300 function.

Chronic inflammation is a key driver of tissue fibrosis and organ aging (López‐Otín et al. 2023). TRPM7 kinase deficiency promotes cardiac fibrosis in mice, leading to heart enlargement and increased stiffness (Rios et al. 2020). Additionally, mice with TRPM7 kinase deficiency exhibit heightened inflammation in the kidneys, spleen, blood vessels, and systemic levels, with elevated expression of pro‐inflammatory factors VCAM1 and IL‐12, as well as collagen deposition in blood vessels—hallmarks of vascular aging (Rios et al. 2020). However, the increased inflammation is likely due to TRPM7 kinase deficiency in immune cells rather than in the organs themselves (Desai et al. 2012; Qiao et al. 2021; Romagnani et al. 2017), as evidence suggests a limited structural impact of TRPM7 kinase on vascular endothelial and smooth muscle function (Wang et al. 2025; Wu et al. 2023). These findings highlight the role of TRPM7 kinase in inflammation and vascular aging, indicating that reduced H3K18 lactylation may not be the sole mechanism by which TRPM7 regulates vascular aging. This underscores the complexity and diversity of TRPM7's role in vascular aging regulation, presenting challenges for fully elucidating its underlying mechanisms.

## 4 Methods

### 4.1 *Trpm7* Knockout (TRPM7ECKO) Mice

*Trpm7* ^fl/fl^ mice were purchased from the Jackson Laboratory. To generate endothelium‐selective deficiency of *Trpm7*, *Trpm7* ^fl/fl^ mice were crossed with Tie2‐Cre mice. Cre‐positive male mice were used for intercrossing to prevent recombination in the female germline. The mice were housed at 20°C ± 2°C with 12 h light/dark cycles and a relative humidity of 50% ± 5% under filtered, pathogen‐free air, with food and water available ad libitum. Animal experiments were performed in accordance with guidelines and protocols approved by the Ethics Committee of Xiangya Hospital (No. 2022020390).

### 4.2 Murine Model of Hind Limb Ischemia (HLI) and Quantifying Microvascular Blood Flow

Perform unilateral femoral artery ligation and excision as a model of experimental PAD. Briefly, a combination of ketamine (Ketathesia; ZooPharm; 90 mg/kg) and xylazine (Anased; Akorn; 10 mg/kg) was injected intraperitoneally (single dose) at 10 μL/g mouse to induce anesthesia. The femoral artery was ligated and resected from just above the inguinal ligament to its bifurcation at the origin of saphenous and popliteal arteries. Perfusion recovery was measured by quantifying microvascular blood flow by laser speckle imaging (Perimed Inc., Ardmore, PA) on days 0, 3, 7, 14, 21, and 28 post‐HLI. Perfusion in the ischemic limb was normalized to a nonischemic limb for each mouse. Mice in each cage were randomly allocated to control or experimental groups. Only the surgeon performing HLI was aware of the treatment groups. Mice were euthanized with an overdose (20 μL/g mouse) of ketamine and xylazine combination followed by cervical dislocation. Organs were isolated and used for subsequent experiments. A minimum of *n* = 4 was used for each group in the experiments according to our previous publications. Mice numbers for each experimental group were indicated in the corresponding figure legend.

### 4.3 Capillary Density

Vascular density was quantified as %CD31+ cells per muscle fiber area on day 21 post‐HLI sections. At least 3 random images per section from each muscle tissue immunostained with CD31 were photographed. In each image, %CD31+ cells per muscle fiber area were counted. Averages of CD31+ cells per muscle fiber area from different groups were plotted on GraphPad Prism 10 and examined for statistical significance.

### 4.4 Immunohistochemistry and Immunofluorescence Analyses

Tissues were isolated from wild‐type and TRPM7ECKO mice, were fixed in 4% paraformaldehyde, and embedded in paraffin. The paraffin blocks were cut into 5 mm thick sections at 30‐mm intervals. The sections from were stained with hematoxylin and eosin, Elastin van Gieson, or antibodies, including anti‐ERG (Abcam, ab92513), anti‐H3K18la (PTM, PTM‐1427RM), anti‐CD31 (R&D, AF3628), anti‐VE‐Cadherin (AiFang, AFRM0013), anti‐vWF (AiFang BIO, AFRM0043), anti‐TRPM7 (Proteintech, 55,251–1‐AP), anti‐MCP1 (Servicebio, GB11199), anti‐collagen‐III (Servicebio, GB111629), anti‐CD45 (Servicebio, GB113885), anti‐MMP‐9 (Servicebio, GB11132), anti‐p300 (affinity, AF5360), anti‐TNF‐α (AiFang, AF11095), and anti‐NF‐κB (AiFang, AFRM0286), followed by species‐specific secondary antibodies. The specimens were observed and imaged under a CQ‐1 microscopy (Yokogawa). Areas of interest were measured using the Image J software.

### 4.5 Cell Culture

Primary HUVECs were gained from ScienCell and cultured in ECM (ScienCell, 1001) supplemented with EC growth supplement (ECGS), 5% fetal bovine serum (FBS), and a penicillin/streptomycin cocktail. HUVECs were used for analysis before the 5th passage.

### 4.6 In Vitro Lentivirus Infection

HUVECs were seeded in 6‐well plates at a density of 1 × 10^5^ cells per well overnight and then transfected with Adenovirus to knockout TRPM7 (Hanheng Biotechnology (Shanghai) Co. Ltd) at an MOI of 50. Three days later, transfection efficiency was determined using a fluorescence microscope (Leica, Germany). After selection with puromycin for 3 days, the cells were used for further experiments. Gene editing efficiency was assessed using the T7 Endonuclease I assay (Vazyme, EN303‐01).

### 4.7 Senescence‐Associated β‐Galactosidase Staining

Metabolic tissues and cells were incubated with an X‐gal mixture (Senescence β‐Galactosidase Staining Kit, C0602, Beyotime) at 37°C for 16 h according to the manufacturer's protocol.

### 4.8 Tube Formation Assay

Tube formation of HUVECs was performed by a μ‐slide angiogenesis (ibidi, 81506). DiI stain (Sigma–Aldrich, 42364) was used for labeling the cell membrane. We precoated the μ‐slide with 10 μL of Matrigel (3432‐005‐01, R&D systems) for 30 min at 37°C. Next, a total of 5 × 10^3^ HUVECs resuspended in 50 μL of the corresponding medium were seeded per well and cultured for 2 h. Four random images for each sample were captured using a microscope (Leica, Germany).

### 4.9 Proliferation Assay

HUVECs proliferation was assayed using an EdU Cell Proliferation Kit (C0071S, Beyotime) according to the manufacturer's instructions. Briefly, HUVECs (5 × 10^3^) were seeded in 96‐well plates and pretreated for 24 h at 37°C with 5% CO_2_. EdU staining was diluted with medium at 1:1000 and added to cells growing in 96‐well plates. After incubation at 37°C with 5% CO_2_ for 2 h, EdU was removed and the cells were washed with PBS at least two times. Next, the cells were fixed with 4% paraformaldehyde for 30 min. Then, the paraformaldehyde was removed. After being washed with PBS three times, 0.5% Triton X‐100 was added and incubated for 15 min. The cells were incubated with click reaction solution for 30 min and washed with 0.5% Triton X‐100 three times. Add 1X Hoechst 33342 solution to each well and incubate at room temperature in the dark for 10 min, then remove and wash with 0.5% Triton X‐100 three times. Four random images for each well were captured and the average value was used for statistical analysis.

### 4.10 Wound Healing Assay

We used an Ibidi culture insert (Ibidi, Germany) to evaluate the migratory ability of different cells. Briefly, HUVECs were seeded into cultured plates according to the manufacturer's protocol and incubated overnight. The next day, the inserts were removed, and media for corresponding treatment conditions were added. Image capture under the microscope at 0, 6, 12, and 24 h, respectively. Image J software was used to calculate the migratory rate.

### 4.11 Cellular Immunofluorescence Staining

Cells were seeded on glass coverslips at 37°C with 5% CO_2_. After removing the cell culture medium and washing with PBS, the cells were fixed with 4% paraformaldehyde for 15 min at room temperature. The cells were washed with 0.03% Triton X‐100 in PBS. Permeabilizations and Block were performed in 0.1% Triton X‐100 in PBS and 3% donkey serum. The cells were stained with primary antibodies at 4°C overnight. After removing primary antibodies and washing with PBST (0.1% Triton X‐100 in PBS), the samples were incubated with Alexa Fluor‐conjugated secondary antibodies (Invitrogen, 1:200) at RT for 1 h. DAPI Fluoromount‐G was used for nuclear counterstaining.

### 4.12 RNA Isolation, Quantitative Real‐Time PCR (RT‐qPCR), and RNA Sequencing

Total RNA was extracted using TRIzol reagent (TaKaRa, 9109). The RNA purity was assessed using the ND‐1000 Nanodrop. Each RNA sample had an A260:A280 ratio above 1.8 and an A260:A230 ratio above 2.0. Complementary DNA (cDNA) was generated using the PrimeScript RT reagent kit (TaKaRa, RR047A) according to the manufacturer's instructions. The cDNA was used for qPCR using TB Green Premix Ex Taq (TaKaRa, RR420A) on a QuantStudio Dx Real‐Time PCR instrument (Thermo Fisher). The reaction was started with a denaturation step at 95°C for 30 s followed by 40 cycles of denaturation at 95°C for 5 s and annealing and elongation at 60°C for 34 s. Fluorescence data were collected at the end of each cycle. The comparative Ct method (△△Ct) was used for analyzing the data. All transcript of genes was corrected by H36B4. The libraries were paired‐end sequenced (PE150, sequencing reads were 150 bp) on an Illumina HiSeq3000 at Novoprotein Co. Ltd. (Suzhou, China). The clean reads were obtained after removal of reads containing adapter, ploy‐N and at low quality from raw data. HISAT2 was used to align the clean reads to the human reference genome hg19 with default parameters. HTSeq was subsequently employed to convert aligned short reads into read counts for each gene model. Differential expression was assessed by DE sequencing read counts as input. The Benjamini‐Hochberg multiple test correction method was enabled. Differentially expressed genes were chosen according to the criteria of *p*‐value < 0.05.

### 4.13 Western Blot Analysis

HUVECs were lysed with ice‐cold RIPA lysis buffer containing protease and phosphatase inhibitors. A bicinchoninic assay (BCA) kit (Beyotime Biotechnology, P0011) was used for protein quantification. Protein in the lysates was separated by SDS‐PAGE and transferred to PVDF membranes (Millipore, IPVH00010). After blocking with 5% nonfat powdered milk (Sangon Biotech, A600669), the membranes were washed with TBST three times and incubated overnight at 4°C with primary antibody. The membranes were incubated with horseradish peroxidase (HRP)‐conjugated secondary antibodies (Abcam, ab6721) for 1 h at room temperature, followed by chemiluminescence detection with Immobilon Crescendo Western HRP substrate (Millipore, WBLUR0500). The imags were generated using ChemiDoc XRS+ (Bio–Rad) with Image Lab software. Band intensities were measured using the Image J software. The antibodies information is below: Anti‐Lamin B1 (PTM BIO, PTM‐5495), Anti‐p16Ink4a (PTM BIO, PTM‐7154), Anti‐p21CIP1(ABclonal, A1483), Anti‐β‐actin (Santa Cruz, Sc‐47778), Anti‐PFKFB3 (abcam, Ab181861), Anti‐PKM2 (Proteintech, 15822–1‐AP), Anti‐LDHA (Proteintech, 19987–1‐AP), Pan‐kla (PTM BIO, PTM‐1401RM), Pan‐kac(PTM BIO, PTM‐105RM), Anti‐Histone H3 (PTM BIO, PTM‐1001RM), Anti‐Histone H4 (PTM BIO, PTM‐1015RM), H3K9la (PTM BIO, PTM‐1419RM), H3K14la (PTM BIO, PTM‐1414RM), H3K18la (PTM BIO, PTM‐1427RM), H3K27la (PTM BIO, PTM‐1428), H4K5la (PTM BIO, PTM‐1407RM), H4K8la (PTM BIO, PTM‐1415RM), H4K12la (PTM BIO, PTM‐1411RM), H4K16la (PTM BIO, PTM‐1417RM), Anti‐ERG (abcam), Anti‐TRPM7 (Proteintech, 55251‐1‐AP), Anti‐CD31 (R&D, AF3628), Anti‐MCP‐1 (Servicebio, GB11199), Anti‐collagen‐III (Servicebio, GB111629), Anti‐CD45 (Servicebio, GB113885), Anti‐MMP‐9 (Servicebio, GB11132), Anti‐γ‐H2A.X (CST, 7631T), Anti‐p300 (Proteintech, 83,078–5‐RR), SUCLG2/GTPSCS (PTM, PTM‐3560).

### 4.14 CUT& Tag

CUT& Tag was performed by Novoprotein Co. Ltd. (Suzhou, China) using the NovoNGS CUT& Tag High‐Sensitivity Kit for Illumina (N259‐YH01‐01A, Novoprotein). Briefly, the HUVECs were bound to concanavalin A‐coated beads. After permeabilization and incubation with primary antibody (anti‐H3K18la, PTM BIO, PTM‐1427RM), the DNA was precisely tagmented with pA‐Tn5 transposase and enriched by PCR to create sequencing‐ready libraries. The library preparations were sequenced using the Illumina Novaseq platform, and 150 bp paired‐end reads were generated.

### 4.15 Quantification and Statistical Analysis

Sample numbers and sizes are indicated by dots. Data are presented as mean ± standard error of the mean (SEM). n values indicated by dots in the figures refer to biological replicates. Quantitative image analysis was conducted using Image J software. In figures and legends, we reported the statistical methods used in all quantitative analyses and precise *P*‐values. Nonparametric tests are preferred. Statistical analyses were conducted using GraphPad Prism 10 (GraphPad Software).

## Author Contributions

Yue Wang, Wanzhou Wu, and Yongping Bai conceived this project and designed the study. Yue Wang and Wanzhou Wu performed and interpreted the experiments. Jing Chen, Xuan Wang, Jin Li, Lingping Zhu, and Shujun Yang performed experiments. Yue Wang and Wanzhou Wu wrote the manuscript. Chuanchang Li and Yongping Bai helped with the proofreading of the manuscript. Yue Wang, Wanzhou Wu, and Zhenyu Li revised the manuscript. All the authors reviewed and approved the manuscript.

## Conflicts of Interest

The authors declare no conflicts of interest.

## Supporting information


**Appendix S1:** acel70244‐sup‐0001‐AppendixS1.docx.

## Data Availability

All data generated or analyzed during this study are available from the corresponding author (or other sources, as applicable) on reasonable request.

## References

[acel70244-bib-0001] Abidin, B. M. , F. J. Rios , A. C. Montezano , and R. M. Touyz . 2025. “Transient Receptor Potential Melastatin 7 Cation Channel, Magnesium and Cell Metabolism in Vascular Health and Disease.” Acta Physiologica 241, no. 2: e14282. 10.1111/apha.14282. 39801180

[acel70244-bib-0002] Baldoli, E. , S. Castiglioni , and J. A. M. Maier . 2013. “Regulation and Function of TRPM7 in Human Endothelial Cells: TRPM7 as a Potential Novel Regulator of Endothelial Function.” PLoS One 8, no. 3: e59891. 10.1371/journal.pone.0059891. 23533657 PMC3606311

[acel70244-bib-0003] Baldoli, E. , and J. A. M. Maier . 2012. “Silencing TRPM7 Mimics the Effects of Magnesium Deficiency in Human Microvascular Endothelial Cells.” Angiogenesis 15, no. 1: 47–57. 10.1007/s10456-011-9242-0. 22183257

[acel70244-bib-0004] Bloom, S. I. , M. T. Islam , L. A. Lesniewski , and A. J. Donato . 2023. “Mechanisms and Consequences of Endothelial Cell Senescence.” Nature Reviews. Cardiology 20, no. 1: 38–51. 10.1038/s41569-022-00739-0. 35853997 PMC10026597

[acel70244-bib-0005] Bowley, G. , S. Irving , I. Hoefer , et al. 2024. “Zebrafish Model for Functional Screening of Flow‐Responsive Genes Controlling Endothelial Cell Proliferation.” Scientific Reports 14, no. 1: 30130. 10.1038/s41598-024-77370-1. 39627337 PMC11615307

[acel70244-bib-0006] Cai, Y. , W. Song , J. Li , et al. 2022. “The Landscape of Aging.” Science China. Life Sciences 65: 2354–2454. 10.1007/s11427-022-2161-3. 36066811 PMC9446657

[acel70244-bib-0007] Chen, J. , J. He , X. Wang , et al. 2025. “Glis1 Inhibits RTEC Cellular Senescence and Renal Fibrosis by Downregulating Histone Lactylation in DKD.” Life Sciences 361: 123293. 10.1016/j.lfs.2024.123293. 39643036

[acel70244-bib-0008] Chen, Y. , L. Liu , L. Xia , et al. 2022. “TRPM7 Silencing Modulates Glucose Metabolic Reprogramming to Inhibit the Growth of Ovarian Cancer by Enhancing AMPK Activation to Promote HIF‐1α Degradation.” Journal of Experimental & Clinical Cancer Research: CR 41, no. 1: 44. 10.1186/s13046-022-02252-1. 35101076 PMC8802454

[acel70244-bib-0009] Clark, K. , M. Langeslag , B. van Leeuwen , et al. 2006. “TRPM7, a Novel Regulator of Actomyosin Contractility and Cell Adhesion.” EMBO Journal 25, no. 2: 290–301. 10.1038/sj.emboj.7600931. 16407977 PMC1383514

[acel70244-bib-0010] Desai, B. N. , G. Krapivinsky , B. Navarro , et al. 2012. “Cleavage of TRPM7 Releases the Kinase Domain From the Ion Channel and Regulates Its Participation in Fas‐Induced Apoptosis.” Developmental Cell 22, no. 6: 1149–1162. 10.1016/j.devcel.2012.04.006. 22698280 PMC3397829

[acel70244-bib-0011] Ding, Y. , Y. Zuo , B. Zhang , et al. 2025. “Comprehensive Human Proteome Profiles Across a 50‐Year Lifespan Reveal Aging Trajectories and Signatures.” Cell 22: S0092‐8674(25)00749‐4. 10.1016/j.cell.2025.06.047. 40713952

[acel70244-bib-0012] Ding, Y.‐N. , X. Tang , H.‐Z. Chen , and D.‐P. Liu . 2018. “Epigenetic Regulation of Vascular Aging and Age‐Related Vascular Diseases.” Advances in Experimental Medicine and Biology 1086: 55–75. 10.1007/978-981-13-1117-8_4. 30232752

[acel70244-bib-0013] Dong, M. , Y. Zhang , M. Chen , et al. 2024. “ASF1A‐Dependent P300‐Mediated Histone H3 Lysine 18 Lactylation Promotes Atherosclerosis by Regulating EndMT.” Acta Pharmaceutica Sinica B 14, no. 7: 3027–3048. 10.1016/j.apsb.2024.03.008. 39027248 PMC11252488

[acel70244-bib-0014] Dorovkov, M. V. , and A. G. Ryazanov . 2004. “Phosphorylation of Annexin I by TRPM7 Channel‐Kinase.” Journal of Biological Chemistry 279, no. 49: 50643–50646. 10.1074/jbc.C400441200. 15485879

[acel70244-bib-0015] Duan, J. , Z. Li , J. Li , et al. 2018. “Structure of the Mammalian TRPM7, a Magnesium Channel Required During Embryonic Development.” Proceedings of the National Academy of Sciences of the United States of America 115, no. 35: E8201–E8210. 10.1073/pnas.1810719115. 30108148 PMC6126765

[acel70244-bib-0016] Fan, Z. , X. Chen , L. Wang , et al. 2023. “LncRNA SNHG8 Regulates the Migration and Angiogenesis of pHUVECs Induced by High Glucose via the TRPM7/ERK1/2 Signaling Axis.” Scientific Reports 13, no. 1: 22485. 10.1038/s41598-023-49779-7. 38110485 PMC10728107

[acel70244-bib-0017] Grunewald, M. , S. Kumar , H. Sharife , et al. 2021. “Counteracting Age‐Related VEGF Signaling Insufficiency Promotes Healthy Aging and Extends Life Span.” Science 373, no. 6554: eabc8479. 10.1126/science.abc8479. 34326210

[acel70244-bib-0018] Harraz, O. F. , and L. J. Jensen . 2020. “Aging, Calcium Channel Signaling and Vascular Tone.” Mechanisms of Ageing and Development 191: 111336. 10.1016/j.mad.2020.111336. 32918949 PMC8511598

[acel70244-bib-0019] He, Y. , G. Yao , C. Savoia , and R. M. Touyz . 2005. “Transient Receptor Potential Melastatin 7 Ion Channels Regulate Magnesium Homeostasis in Vascular Smooth Muscle Cells: Role of Angiotensin II.” Circulation Research 96, no. 2: 207–215. 10.1161/01.RES.0000152967.88472.3e. 15591230

[acel70244-bib-0020] Hernandez‐Segura, A. , J. Nehme , and M. Demaria . 2018. “Hallmarks of Cellular Senescence.” Trends in Cell Biology 28, no. 6: 436–453. 10.1016/j.tcb.2018.02.001. 29477613

[acel70244-bib-0021] Hu, J. , M. S. Leisegang , M. Looso , et al. 2023. “Disrupted Binding of Cystathionine γ‐Lyase to p53 Promotes Endothelial Senescence.” Circulation Research 133: 842–857. 10.1161/CIRCRESAHA.123.323084. 37800327

[acel70244-bib-0022] Huang, X. , X. Zhang , N. Machireddy , et al. 2023. “Endothelial FoxM1 Reactivates Aging‐Impaired Endothelial Regeneration for Vascular Repair and Resolution of Inflammatory Lung Injury.” Science Translational Medicine 15, no. 709: eabm5755. 10.1126/scitranslmed.abm5755. 37585502 PMC10894510

[acel70244-bib-0023] Inoue, K. , and Z.‐G. Xiong . 2009. “Silencing TRPM7 Promotes Growth/Proliferation and Nitric Oxide Production of Vascular Endothelial Cells via the ERK Pathway.” Cardiovascular Research 83, no. 3: 547–557. 10.1093/cvr/cvp153. 19454490 PMC2709465

[acel70244-bib-0024] Ji, Y. , Z. Xu , L. Tang , et al. 2025. “O‐GlcNAcylation of YBX1 Drives a Glycolysis‐Histone Lactylation Feedback Loop in Hepatocellular Carcinoma.” Cancer Letters 631: 217957. 10.1016/j.canlet.2025.217957. 40721081

[acel70244-bib-0025] Jiang, J. , M. H. Li , K. Inoue , X. P. Chu , J. Seeds , and Z. G. Xiong . 2007. “Transient Receptor Potential Melastatin 7‐Like Current in Human Head and Neck Carcinoma Cells: Role in Cell Proliferation.” Cancer Research 67, no. 22: 10929–10938. 10.1158/0008-5472.CAN-07-1121. 18006838 PMC2398732

[acel70244-bib-0026] Jiang, M. , Z. Zheng , X. Wang , et al. 2024. “A Biomarker Framework for Liver Aging: The Aging Biomarker Consortium Consensus Statement.” Lifestyle Medicine 3, no. 1: lnae004. 10.1093/lifemedi/lnae004. PMC11749002 39872390

[acel70244-bib-0027] Krapivinsky, G. , L. Krapivinsky , Y. Manasian , and D. E. Clapham . 2014. “The TRPM7 Chanzyme Is Cleaved to Release a Chromatin‐Modifying Kinase.” Cell 157, no. 5: 1061–1072. 10.1016/j.cell.2014.03.046. 24855944 PMC4156102

[acel70244-bib-0028] Krapivinsky, G. , S. Mochida , L. Krapivinsky , S. M. Cibulsky , and D. E. Clapham . 2006. “The TRPM7 Ion Channel Functions in Cholinergic Synaptic Vesicles and Affects Transmitter Release.” Neuron 52, no. 3: 485–496. 17088214 10.1016/j.neuron.2006.09.033

[acel70244-bib-0029] Lacolley, P. , V. Regnault , P. Segers , and S. Laurent . 2017. “Vascular Smooth Muscle Cells and Arterial Stiffening: Relevance in Development, Aging, and Disease.” Physiological Reviews 97, no. 4: 1555–1617. 10.1152/physrev.00003.2017. 28954852

[acel70244-bib-0030] Li, X. , M. Chen , X. Chen , et al. 2024. “TRAP1 Drives Smooth Muscle Cell Senescence and Promotes Atherosclerosis via HDAC3‐Primed Histone H4 Lysine 12 Lactylation.” European Heart Journal 45, no. 39: 4219–4235. 10.1093/eurheartj/ehae379. 39088352 PMC11481199

[acel70244-bib-0031] Li, X. , Q. Wang , J. Fei , et al. 2025. “Lactate Promotes Premature Aging of Preeclampsia Placentas Through Histone Lactylation‐Regulated GADD45A.” Placenta 161: 39–51. 10.1016/j.placenta.2025.01.010. 39908745

[acel70244-bib-0032] Li, X. , Y. Yang , B. Zhang , et al. 2022. “Lactate Metabolism in Human Health and Disease.” Signal Transduction and Targeted Therapy 7, no. 1: 305. 10.1038/s41392-022-01151-3. 36050306 PMC9434547

[acel70244-bib-0033] Liu, M. , L. Gu , Y. Zhang , et al. 2024. “LKB1 Inhibits Telomerase Activity Resulting in Cellular Senescence Through Histone Lactylation in Lung Adenocarcinoma.” Cancer Letters 595: 217025. 10.1016/j.canlet.2024.217025. 38844063

[acel70244-bib-0034] Liu, R. , X. Ren , Y. E. Park , et al. 2025. “Nuclear GTPSCS Functions as a Lactyl‐CoA Synthetase to Promote Histone Lactylation and Gliomagenesis.” Cell Metabolism 37, no. 2: 377–394. 10.1016/j.cmet.2024.11.005. 39642882 PMC11798710

[acel70244-bib-0035] López‐Otín, C. , M. A. Blasco , L. Partridge , M. Serrano , and G. Kroemer . 2023. “Hallmarks of Aging: An Expanding Universe.” Cell 186, no. 2: 243–278. 10.1016/j.cell.2022.11.001. 36599349

[acel70244-bib-0036] Maus, M. , V. López‐Polo , L. Mateo , et al. 2023. “Iron Accumulation Drives Fibrosis, Senescence and the Senescence‐Associated Secretory Phenotype.” Nature Metabolism 5, no. 12: 2111–2130. 10.1038/s42255-023-00928-2. PMC10730403 38097808

[acel70244-bib-0037] Mehdizadeh, M. , M. Aguilar , E. Thorin , G. Ferbeyre , and S. Nattel . 2022. “The Role of Cellular Senescence in Cardiac Disease: Basic Biology and Clinical Relevance.” Nature Reviews. Cardiology 19, no. 4: 250–264. 10.1038/s41569-021-00624-2. 34667279

[acel70244-bib-0038] Mojiri, A. , B. K. Walther , C. Jiang , et al. 2021. “Telomerase Therapy Reverses Vascular Senescence and Extends Lifespan in Progeria Mice.” European Heart Journal 42, no. 42: 4352–4369. 10.1093/eurheartj/ehab547. 34389865 PMC8603239

[acel70244-bib-0039] Nadler, M. J. , M. C. Hermosura , K. Inabe , et al. 2001. “LTRPC7 Is a Mg.ATP‐Regulated Divalent Cation Channel Required for Cell Viability.” Nature 411, no. 6837: 590–595. 11385574 10.1038/35079092

[acel70244-bib-0040] Pinto, M. , A. M. Pickrell , X. Wang , et al. 2017. “Transient Mitochondrial DNA Double Strand Breaks in Mice Cause Accelerated Aging Phenotypes in a ROS‐Dependent but p53/p21‐Independent Manner.” Cell Death and Differentiation 24, no. 2: 288–299. 10.1038/cdd.2016.123. 27911443 PMC5299712

[acel70244-bib-0041] Qiao, W. , K. H. M. Wong , J. Shen , et al. 2021. “TRPM7 Kinase‐Mediated Immunomodulation in Macrophage Plays a Central Role in Magnesium Ion‐Induced Bone Regeneration.” Nature Communications 12, no. 1: 2885. 10.1038/s41467-021-23005-2. PMC8128914 34001887

[acel70244-bib-0042] Rios, F. J. , Z. G. Zou , A. P. Harvey , et al. 2020. “Chanzyme TRPM7 Protects Against Cardiovascular Inflammation and Fibrosis.” Cardiovascular Research 116, no. 3: 721–735. 10.1093/cvr/cvz164. 31250885 PMC7252442

[acel70244-bib-0043] Romagnani, A. , V. Vettore , T. Rezzonico‐Jost , et al. 2017. “TRPM7 Kinase Activity Is Essential for T Cell Colonization and Alloreactivity in the Gut.” Nature Communications 8, no. 1: 1917. 10.1038/s41467-017-01960-z. PMC5714948 29203869

[acel70244-bib-0044] Rössig, A. , K. Hill , W. Nörenberg , et al. 2022. “Pharmacological Agents Selectively Acting on the Channel Moieties of TRPM6 and TRPM7.” Cell Calcium 106: 102640. 10.1016/j.ceca.2022.102640. 36030694

[acel70244-bib-0045] Runnels, L. W. , L. Yue , and D. E. Clapham . 2001. “TRP‐PLIK, a Bifunctional Protein With Kinase and Ion Channel Activities.” Science 291, no. 5506: 1043–1047. 11161216 10.1126/science.1058519

[acel70244-bib-0046] Schmitz, C. , A. L. Perraud , C. O. Johnson , et al. 2003. “Regulation of Vertebrate Cellular Mg^2+^ Homeostasis by TRPM7.” Cell 114, no. 2: 191–200. 10.1016/s0092-8674(03)00556-7. 12887921

[acel70244-bib-0047] Ungvari, Z. , S. Tarantini , T. Kiss , et al. 2018. “Endothelial Dysfunction and Angiogenesis Impairment in the Ageing Vasculature.” Nature Reviews. Cardiology 15, no. 9: 555–565. 10.1038/s41569-018-0030-z. 29795441 PMC6612360

[acel70244-bib-0048] Uryga, A. , K. Gray , and M. Bennett . 2016. “DNA Damage and Repair in Vascular Disease.” Annual Review of Physiology 78: 45–66. 10.1146/annurev-physiol-021115-105127. 26442438

[acel70244-bib-0049] Venkatachalam, K. 2022. “Regulation of Aging and Longevity by Ion Channels and Transporters.” Cells 11, no. 7: 1180. 10.3390/cells11071180. 35406743 PMC8997527

[acel70244-bib-0050] Wang, X. , M. Wang , T.‐T. Zhu , et al. 2025. “The TRPM7 Chanzyme in Smooth Muscle Cells Drives Abdominal Aortic Aneurysm in Mice.” Nature Cardiovascular Research 4, no. 2: 216–234. 10.1038/s44161-025-00613-5. 39953275

[acel70244-bib-0051] Wu, W. , X. Wang , L. Liao , et al. 2023. “The TRPM7 Channel Reprograms Cellular Glycolysis to Drive Tumorigenesis and Angiogenesis.” Cell Death & Disease 14, no. 3: 183. 10.1038/s41419-023-05701-7. 36878949 PMC9988972

[acel70244-bib-0052] Yankaskas, C. L. , K. Bera , K. Stoletov , et al. 2021. “The Fluid Shear Stress Sensor TRPM7 Regulates Tumor Cell Intravasation.” Science Advances 7, no. 28: eabh3457. 10.1126/sciadv.abh3457. 34244134 PMC8270498

[acel70244-bib-0053] Zeng, Z. , K. Inoue , H. Sun , et al. 2015. “TRPM7 Regulates Vascular Endothelial Cell Adhesion and Tube Formation.” American Journal of Physiology. Cell Physiology 308, no. 4: C308–C318. 10.1152/ajpcell.00275.2013. 25472964 PMC4329423

[acel70244-bib-0054] Zhang, D. , Z. Tang , H. Huang , et al. 2019. “Metabolic Regulation of Gene Expression by Histone Lactylation.” Nature 574, no. 7779: 575–580. 10.1038/s41586-019-1678-1. 31645732 PMC6818755

[acel70244-bib-0055] Zhang, J. , X. Wang , Z. Fu , et al. 2024. “Long‐Term Simulated Microgravity Fosters Carotid Aging‐Like Changes via Piezo1.” Cardiovascular Research 120, no. 5: 548–559. 10.1093/cvr/cvae024. 38271270

[acel70244-bib-0056] Zhou, D.‐M. , L.‐L. Sun , J. Zhu , B. Chen , X.‐Q. Li , and W.‐D. Li . 2020. “MiR‐9 Promotes Angiogenesis of Endothelial Progenitor Cell to Facilitate Thrombi Recanalization via Targeting TRPM7 Through PI3K/Akt/Autophagy Pathway.” Journal of Cellular and Molecular Medicine 24, no. 8: 4624–4632. 10.1111/jcmm.15124. 32147957 PMC7176881

[acel70244-bib-0057] Zhu, D. , J. You , N. Zhao , and H. Xu . 2019. “Magnesium Regulates Endothelial Barrier Functions Through TRPM7, MagT1, and S1P1.” Advanced Science 6, no. 18: 1901166. 10.1002/advs.201901166. 31559137 PMC6755513

