# Supplementary material for: TRPM7 Deficiency Accelerates Vascular Senescence by Inhibiting H3K18 Lactylation
Source: Aging Cell. 2025 Oct 2;24(11):e70244. doi: 10.1111/acel.70244 (PMC12611325; doi:10.1111/acel.70244)
Supplement: Supplementary file 1 — Appendix S1: acel70244‐sup‐0001‐AppendixS1.docx. [file ACEL-24-e70244-s001.docx]

**
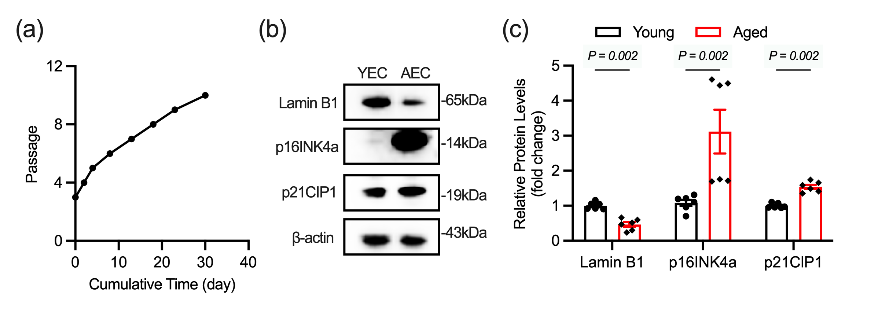
Supplementary Figure S1.** Construction of a senescent HUVEC model via serial passaging. (a) Serial passaging affects the population doubling time of HUVECs. (b-c) Changes in senescence-associated genes in aged endothelial cells (AEC, p10) compared to young endothelial cells (YEC, p3). Differences in (c) are tested using unpaired multiple Mann-Whitney tests.

**Supplementary Figure S2.**
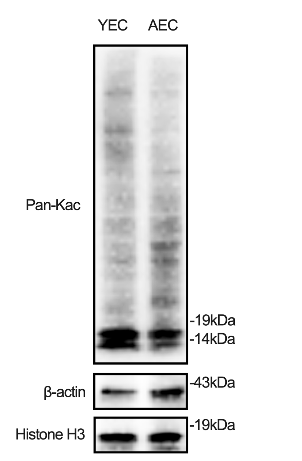
 Aging has a limited impact on protein pan-acetylation in HUVECs.

**Supplementary Figure S3.
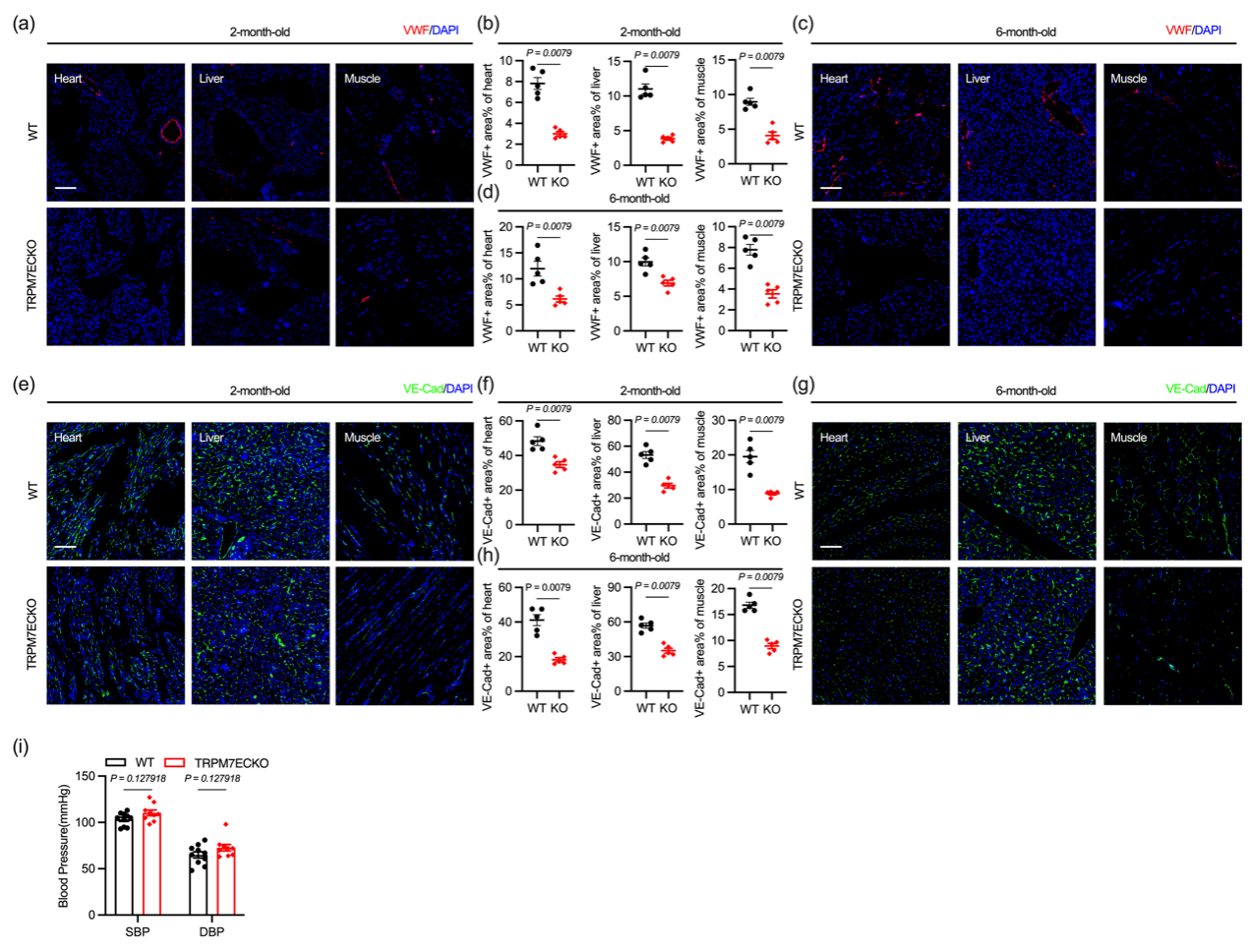
** TRPM7ECKO mice exhibit altered blood vessel density in multiple organs and changes in blood pressure. (a-h) Immunofluorescence staining for vWF and VE-Cadherin was used to further label microvascular endothelium in tissues. (i) Endothelial TRPM7 deletion does not affect blood pressure in 6-month-old mice. Scale bar: 200 μm in (a-g) All differences in are tested using unpaired multiple Mann-Whitney tests.

**
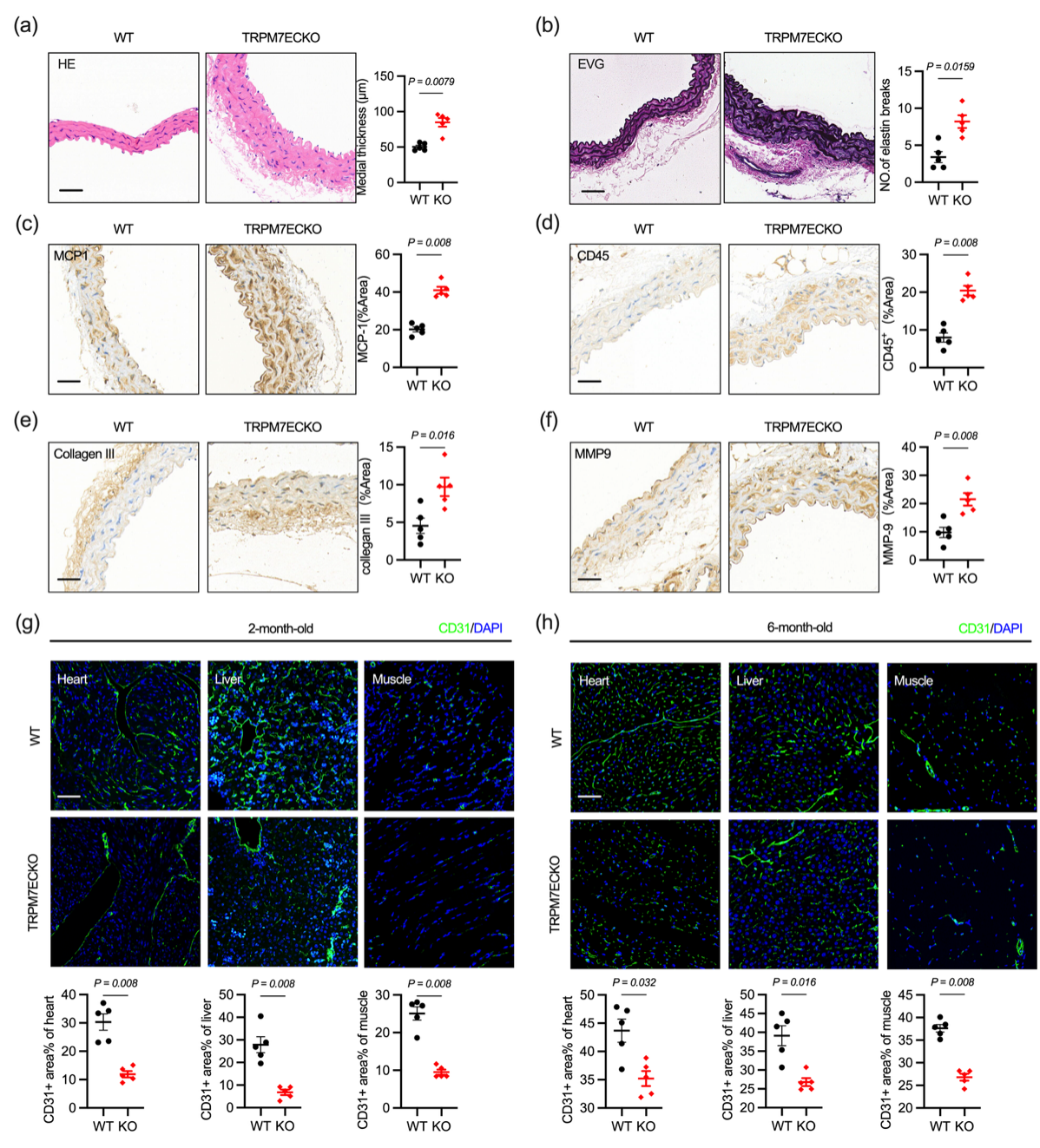
Supplementary Figure S4.** TRPM7ECKO female mice exhibit accelerated vascular aging. (a-b) HE and EVG staining for mice aorta. (c-d) MCP1 and CD45 levels are elevated in the aortas of female TRPM7ECKO mice. (e-f) TRPM7ECKO female mice exhibit increased collagen III and MMP9 expression in the aorta. (g-h) TRPM7ECKO female mice exhibit reduced vascular endothelial coverage in the heart, liver, and muscle. Scale bar: 80 μm in (a-b), 40 μm in (c-f), 200 μm in (g-h). All differences in this figure are tested using Mann-Whitney tests.

**
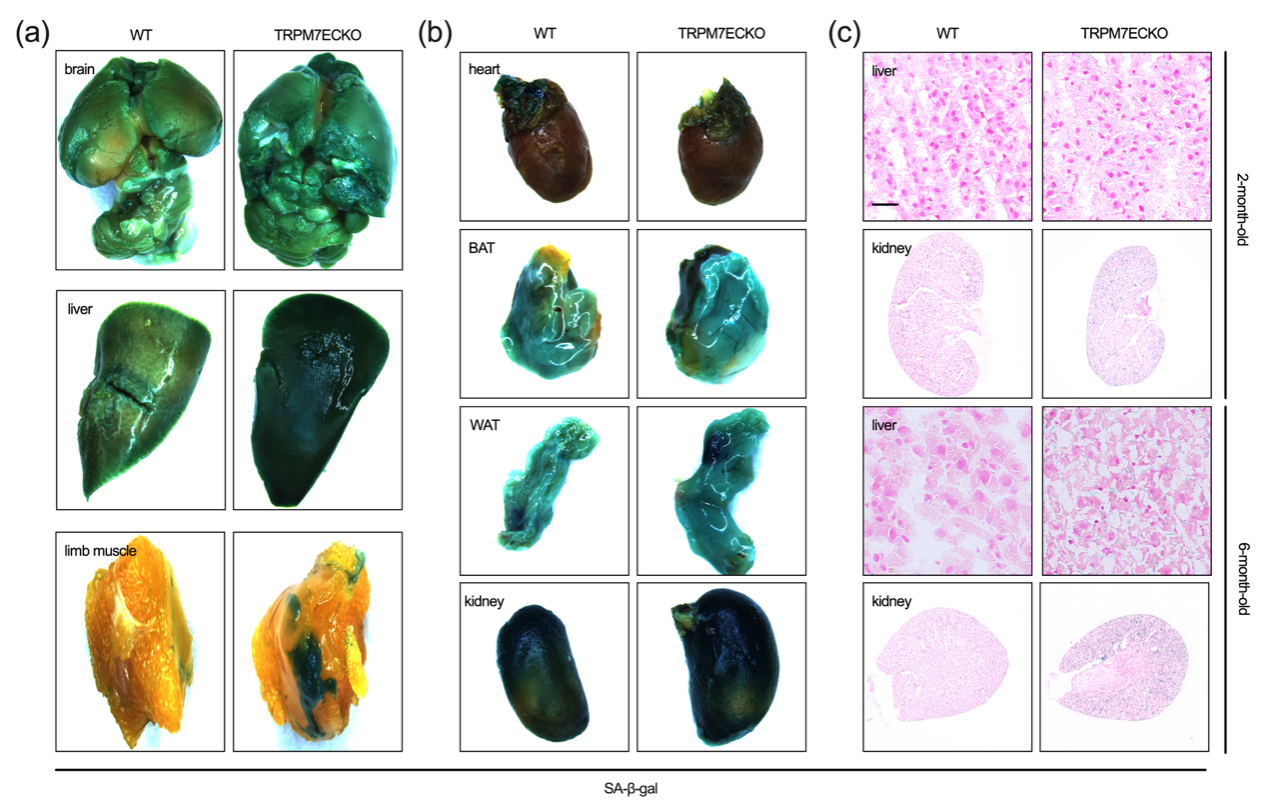
Supplementary Figure S5.** Assessment of multi-organ aging in TRPM7ECKO male mice. (a-b) SA-β-gal staining was performed on the brain, liver, lower limb muscle, heart, brown adipose tissue (BAT), white adipose tissue (WAT), and kidneys of 6-month-old mice. (c) SA-β-gal staining was performed on frozen liver and kidney sections from 2-month-old and 6-month-old male mice. Scale bar: 400 μm in (c).

**
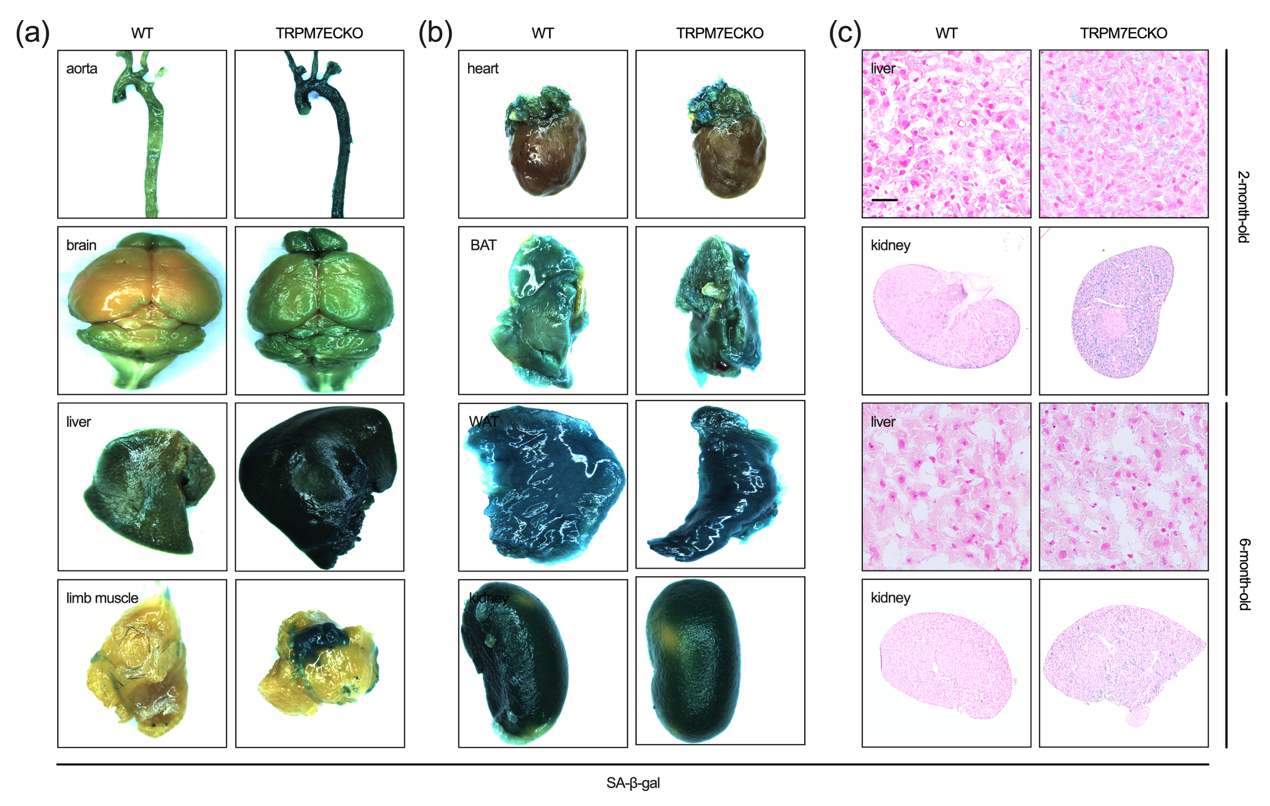
Supplementary Figure S6.** Assessment of multi-organ aging in TRPM7ECKO female mice. (a-b) SA-β-gal staining was performed on the aorta, brain, liver, lower limb muscle, heart, brown adipose tissue (BAT), white adipose tissue (WAT), and kidneys of 6-month-old female mice. (c) SA-β-gal staining was performed on frozen liver and kidney sections from 2-month-old and 6-month-old female mice. Scale bar: 400 μm in (c).

**
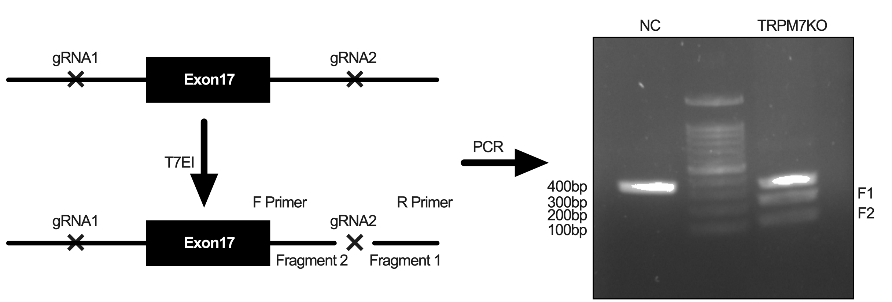
Supplementary Figure S7.** TRPM7 knockout HUVECs validation.

**Supplementary Figure S8.** Pharmacological inhibition of TRPM7 reduces histone H3K18 lactylation in endothelial cells.**
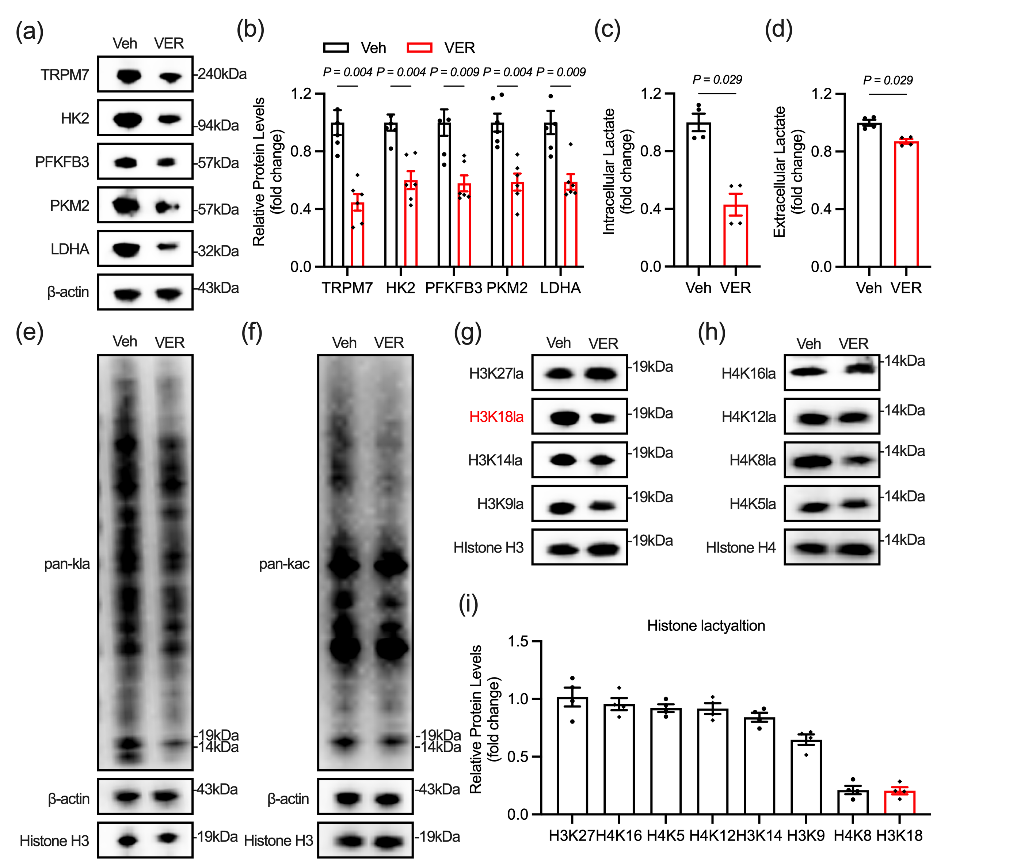
** (a-b) VER155008 inhibits TRPM7, leading to decreased protein levels of glycolytic enzymes in HUVECs. (c-d) VER155008 inhibits TRPM7 and reduces lactate production in HUVECs. (e-f) Pharmacological inhibition of TRPM7 impacts protein lactylation and acetylation. (g-i) Histone H3K18 lactylation was the most significantly impaired modification in HUVECs treated with VER155008. All differences in this figure are tested using Mann-Whitney tests.

**
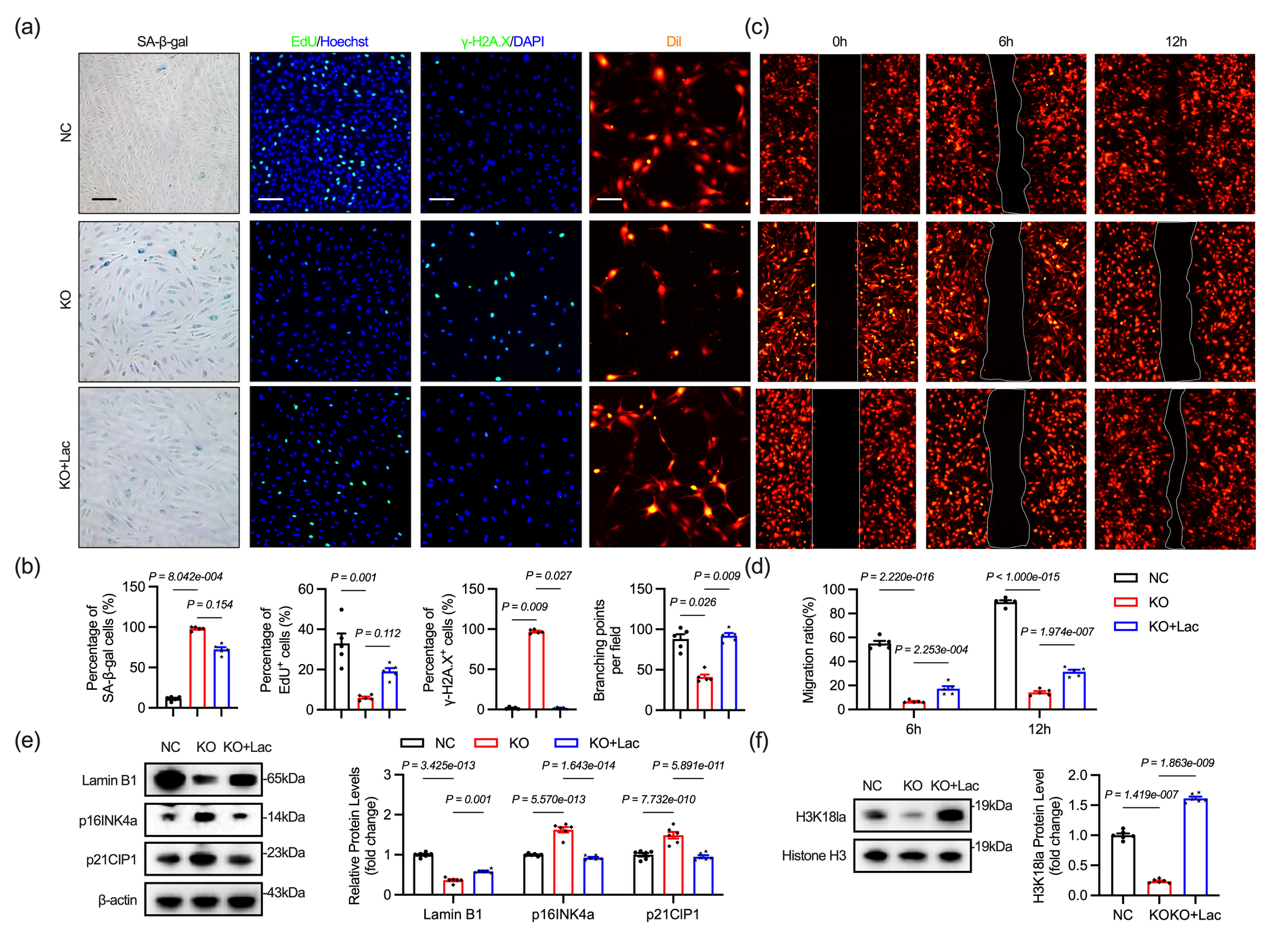
Supplementary Figure S9.** Lactate rescues TRPM7-deficient endothelial cells from premature senescence. (a-d) Lactate supplementation (10 mM, 24 hours) rescues premature senescence and dysfunction in TRPM7KO endothelial cells. (e) Lactate supplementation alters senescence genes in TRPM7KO endothelial cells. (f) Lactate addition increases histone H3K18 lactylation. Scale bar: 300 μm in (c), 300 μm in SA-β-gal stanning and 200 μm in EdU and γ-H2A.X and 120 μm in tubeformation. Differences in (b, f) are tested using Mann-Whitney tests and (d, e) are using 2-way ANOVA with Dunnett’s correction.


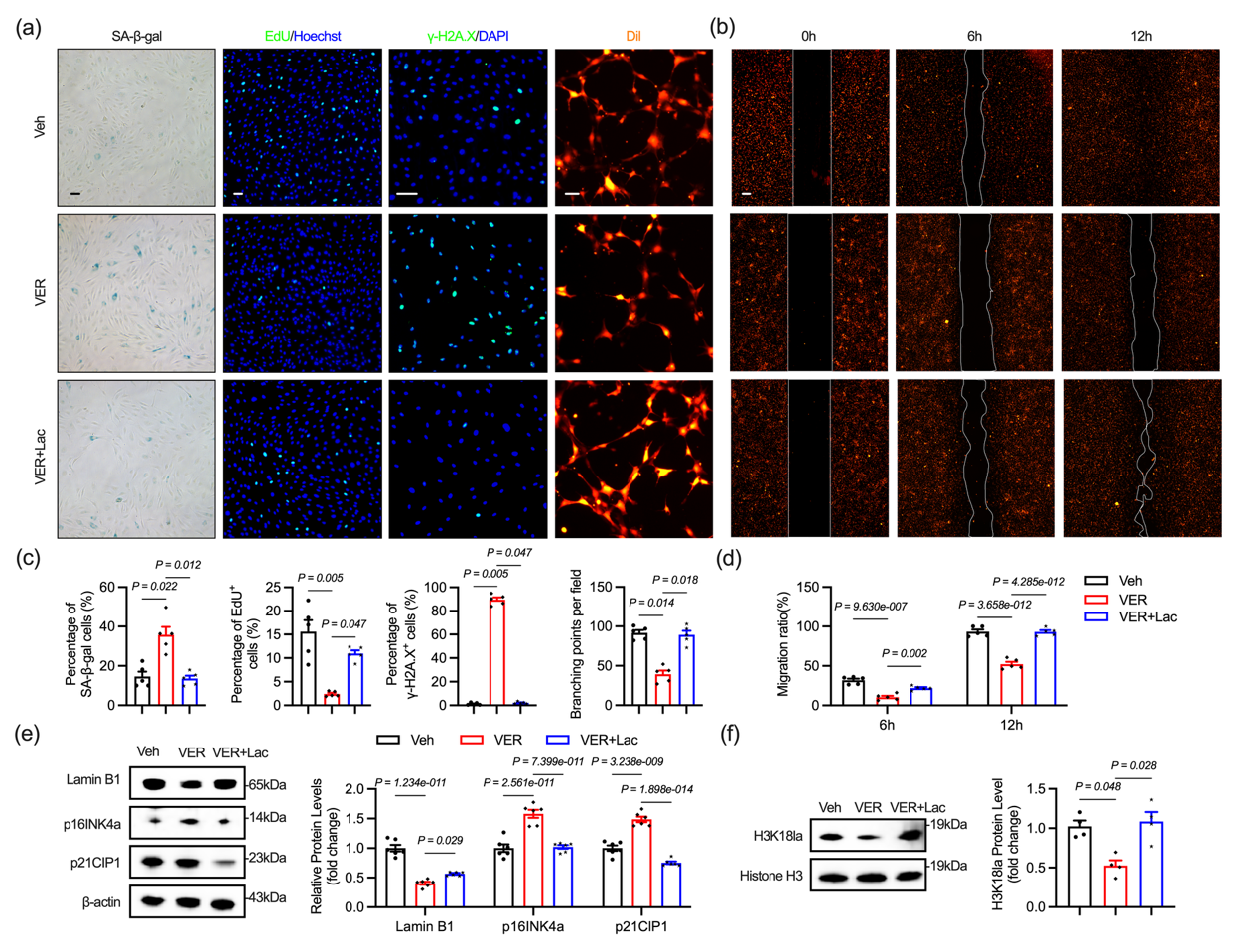
**Supplementary Figure S10.** Lactate supplementation rescued endothelial cells senescence induced by TRPM7 inhibitor. (a-d) Lactate supplementation (10 mM, 24 hours) rescues premature senescence and dysfunction in TRPM7 inhibitor incubated ECs. (e) Lactate modulates TRPM7 inhibitor-induced changes in endothelial senescence gene expression. (f) Lactate supplementation rescued the decrease in H3K18 lactylation caused by TRPM7 inhibition. Scale bar: 300 μm in (b), 300 μm in SA-β-gal stanning and 200 μm in EdU and γ-H2A.X and 120 μm in tubeformation. Differences in (b, f) are tested using Mann-Whitney tests and (d, e) are using 2-way ANOVA with Dunnett’s correction.

**
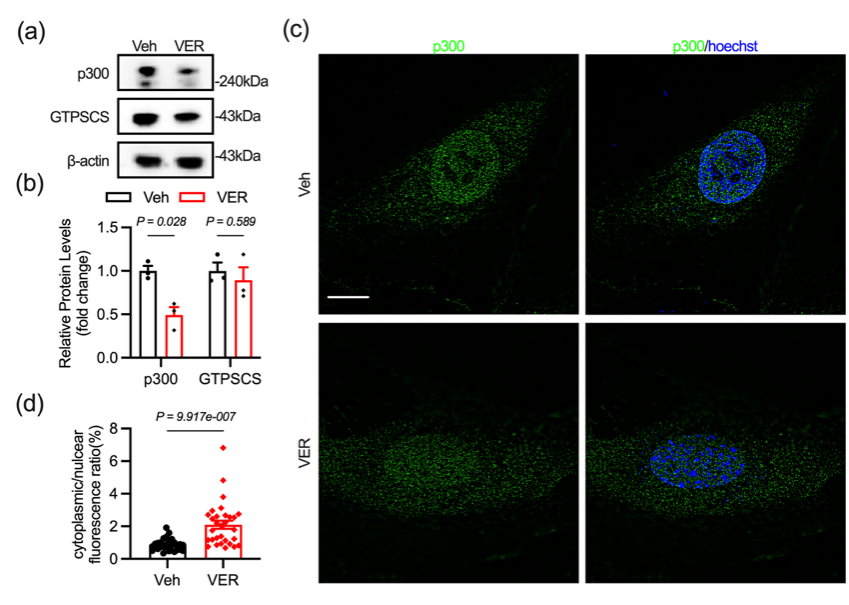
Supplementary Figure S11.** Pharmacological inhibition of TRPM7 reduces p300 protein levels and nuclear localization. (a-b) TRPM7 inhibitor reduced p300 protein but not GTPSCS in vitro. (c-d) TRPM7 inhibitors decrease the ratio of p300 nuclear localization signal. Differences in (d) are tested using Mann-Whitney tests and (b) are using multiple unpaired Welch t-test.

**
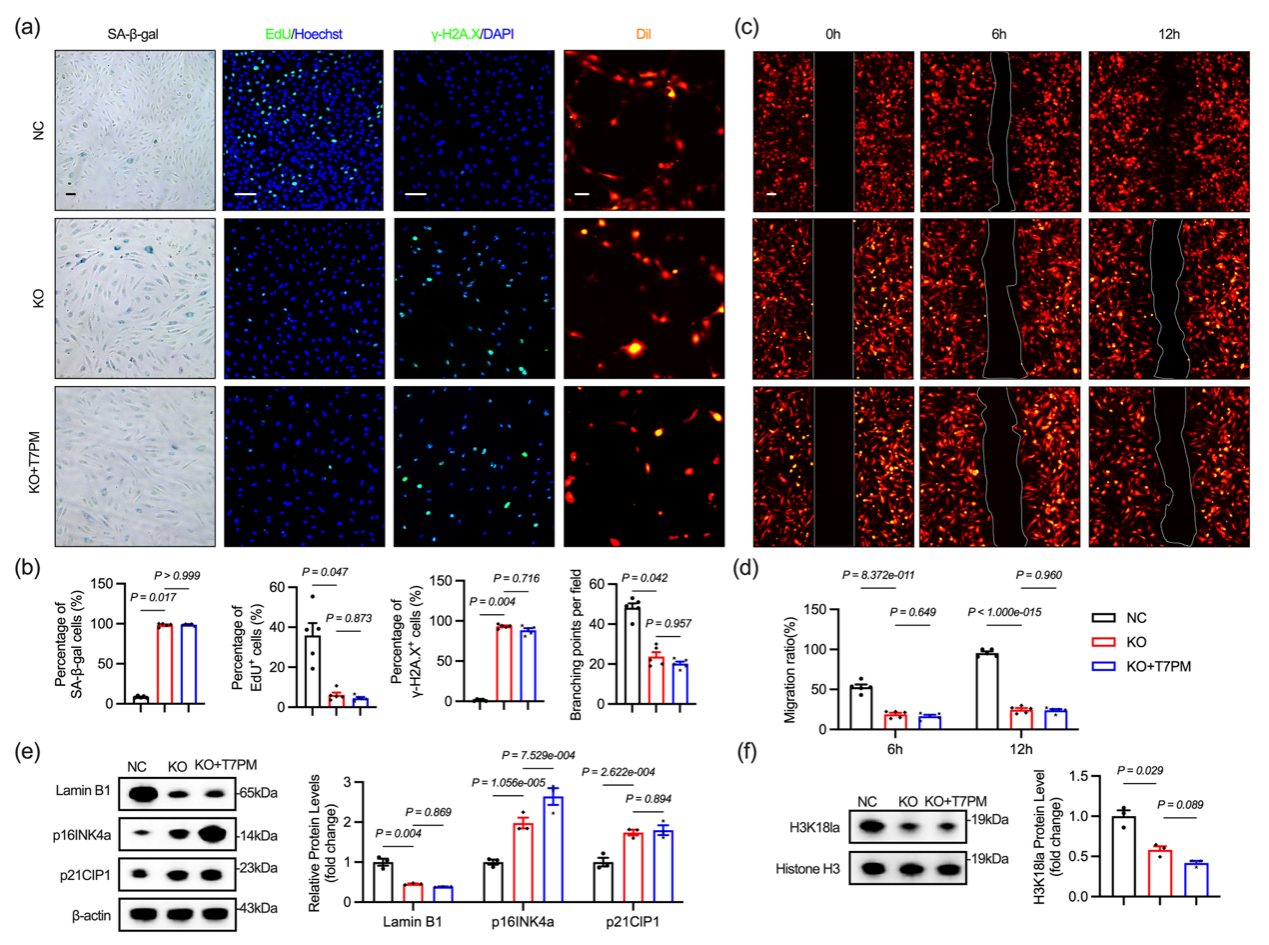
Supplementary Figure S12.** TRPM7 channel loss-of-function mutants fail to rescue endothelial senescence caused by TRPM7 deficiency. (a-d) Transfection of TRPM7 channel pore mutants (AA 1090-1092 NLL, T7PM) did not affect the aging phenotypes of TRPM7-deficient endothelial cells. (e) Transfection of T7PM did not reverse the aging-related gene expression changes observed in TRPM7KO endothelial cells. (f) T7PM did not affect histone H3K18 lactylation. Scale bar: 300 μm in (c), 300 μm in SA-β-gal stanning and 200 μm in EdU and γ-H2A.X and 120 μm in tubeformation. Differences in (b, f) are tested using Mann-Whitney tests and (d, e) are using 2-way ANOVA with Dunnett’s correction.

**Supplementary Figure S13.
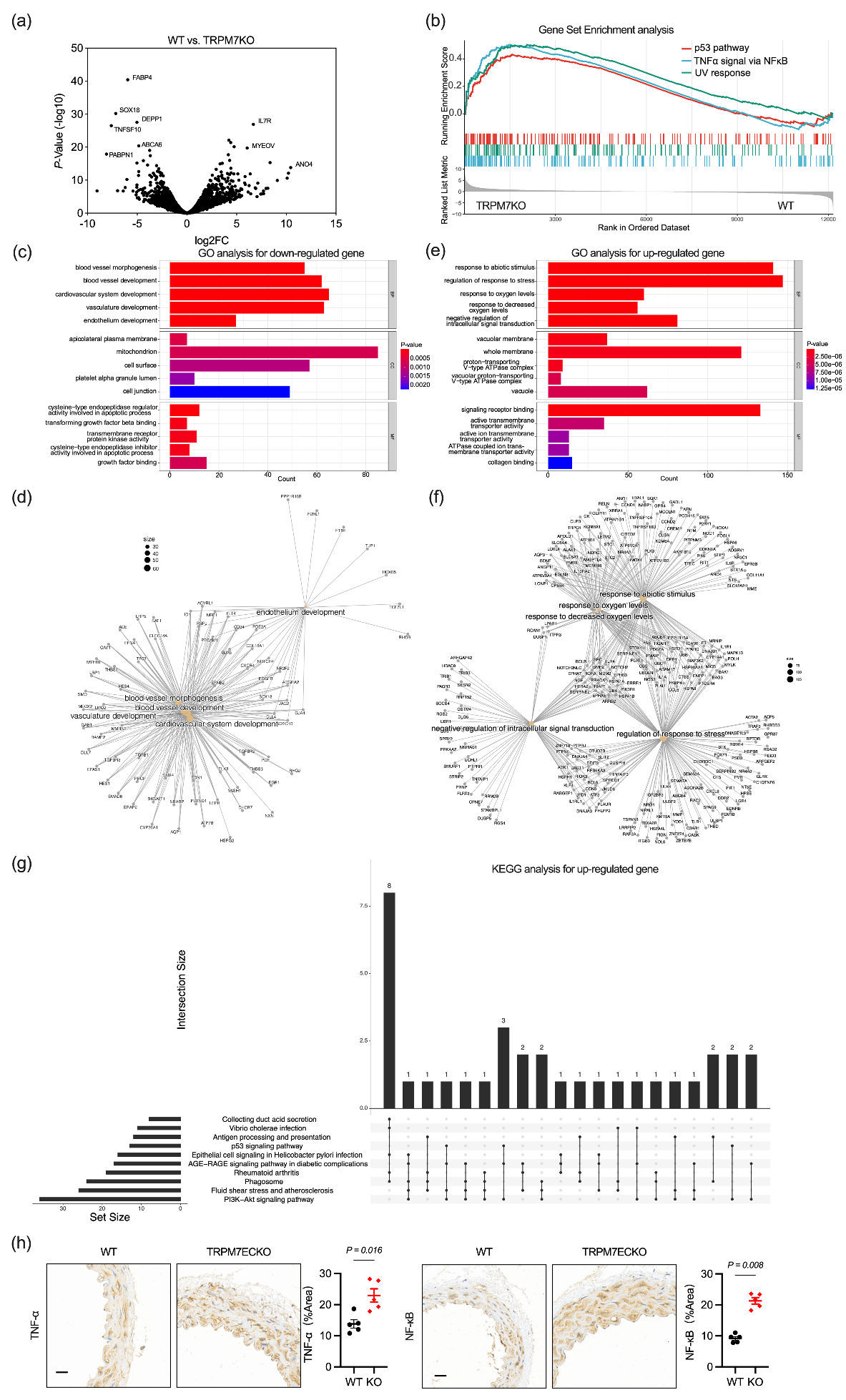
** Transcriptomics reveals transcriptional alterations in TRPM7-deficient endothelial cells. (a) TRPM7KO endothelial cells exhibit differentially expressed genes compared to WT. (b) Gene Set Enrichment Analysis (GSEA) for RNA-seq. (c-g) GO and KEGG analysis for differential up or down genes. (h) Expression of TNF and NF-κB in the aorta of TRPM7 endothelial knockout mice. Scale bar: 40 μm in (h). All differences in this figure are tested using Mann-Whitney tests.

**
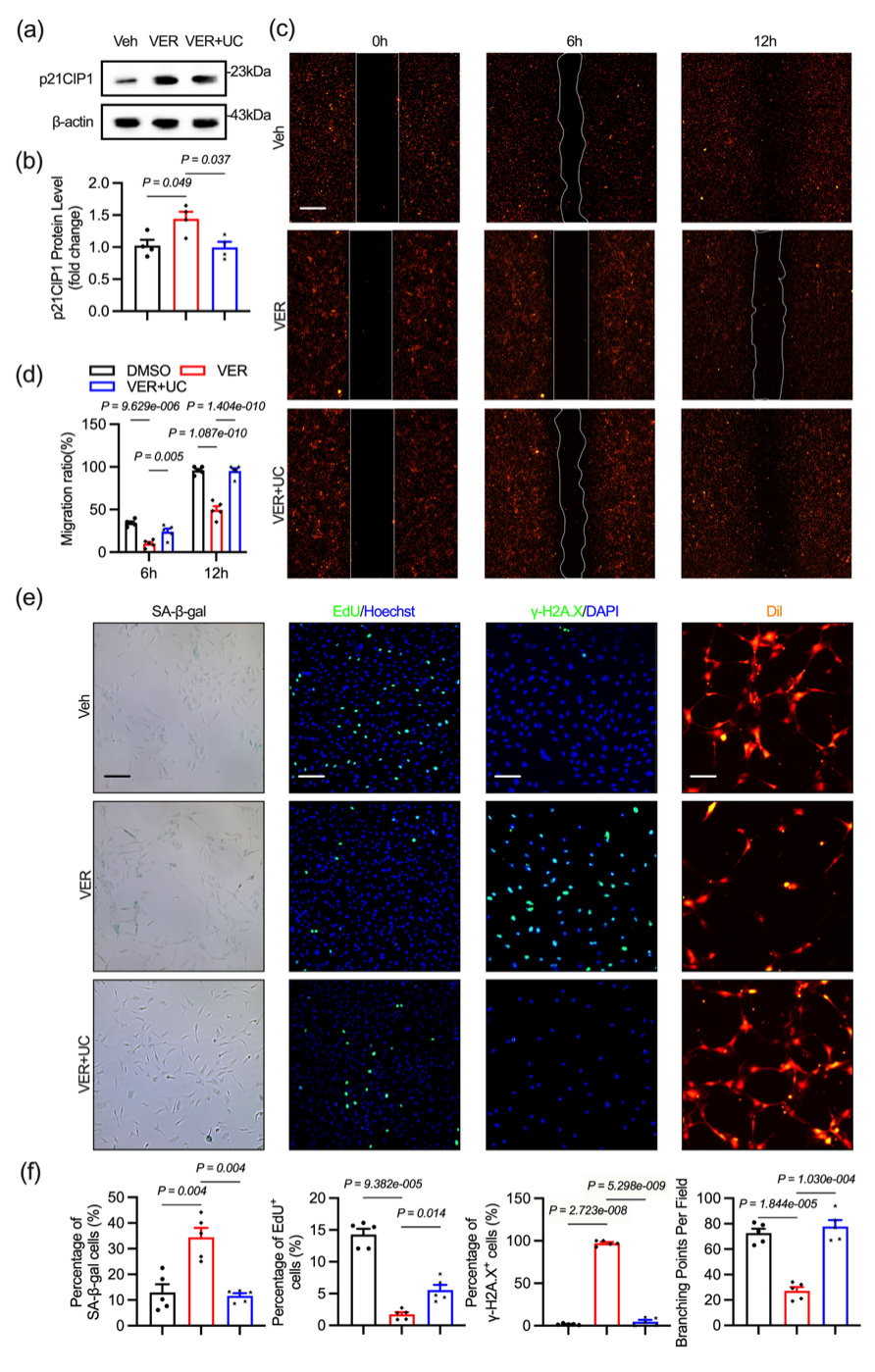
Supplementary Figure S14.** Pharmacological inhibition of p21CIP1 attenuates premature senescence in ECs induced by TRPM7 inhibitor. (a-b) UC2288 (10 μM, 24 hours) counteracts p21 rise in TRPM7 inhibitor incubated ECs. (c-f) UC2288 mitigates premature senescence and dysfunction in TRPM7 inhibitor incubated ECs. Scale bar: 300 μm in (c), 300 μm in SA-β-gal stanning and 200 μm in EdU and γ-H2A.X and 120 μm in tubeformation. Differences in (c) are tested using Kruskal-Wallis test with Dunn’s correction and (e, g) are using Brown-Forsythe and Welch ANOVA tests.
